# Neurotoxic Effects of Mixtures of Perfluoroalkyl Substances (PFAS) at Environmental and Human Blood Concentrations

**DOI:** 10.1021/acs.est.4c06017

**Published:** 2024-09-11

**Authors:** Karla M. Ríos-Bonilla, Diana S. Aga, Jungeun Lee, Maria König, Weiping Qin, Judith R. Cristobal, Gunes Ekin Atilla-Gokcumen, Beate I. Escher

**Affiliations:** † Department of Chemistry, University at Buffalo - The State University of New York, Buffalo, New York 14260, United States; ‡ Department of Cell Toxicology, Helmholtz-Centre for Environmental Research − UFZ, Leipzig 04318, Germany

**Keywords:** PFAS, mixtures, neurotoxicity, mitochondrial toxicity, oxidative stress, environmental monitoring, AREc32

## Abstract

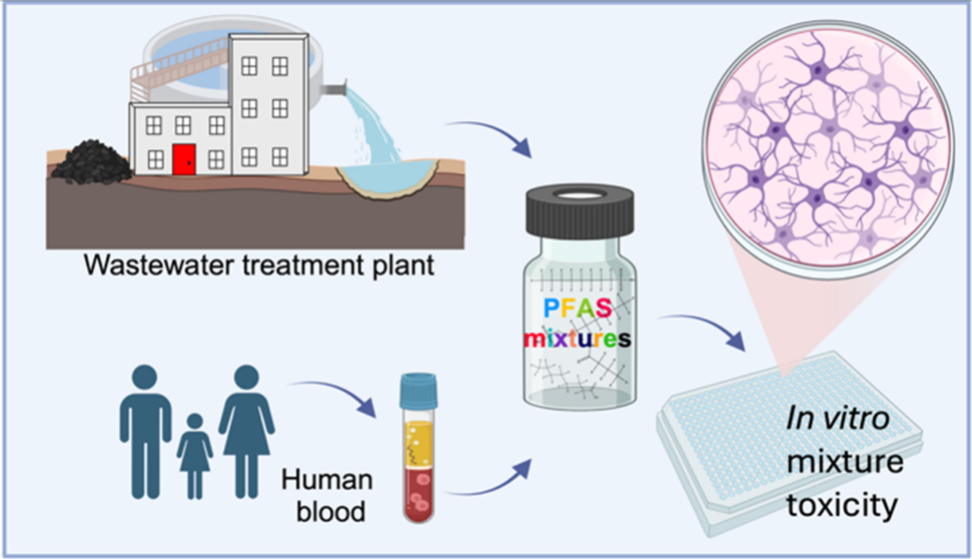

Per- and polyfluoroalkyl substances (PFAS) may cause various deleterious health effects. Epidemiological studies have demonstrated associations between PFAS exposure and adverse neurodevelopmental outcomes. The cytotoxicity, neurotoxicity, and mitochondrial toxicity of up to 12 PFAS including perfluoroalkyl carboxylates, perfluoroalkyl sulfonates, 6:2 fluorotelomer sulfonic acid (6:2 FTSA), and hexafluoropropylene oxide-dimer acid (HPFO-DA) were tested at concentrations typically observed in the environment (e.g., wastewater, biosolids) and in human blood using high-throughput *in vitro* assays. The cytotoxicity of all individual PFAS was classified as baseline toxicity, for which prediction models based on partition constants of PFAS between biomembrane lipids and water exist. No inhibition of the mitochondrial membrane potential and activation of oxidative stress response were observed below the cytotoxic concentrations of any PFAS tested. All mixture components and the designed mixtures inhibited the neurite outgrowth in differentiated neuronal cells derived from the SH-SY5Y cell line at concentrations around or below cytotoxicity. All designed mixtures acted according to concentration addition at low effect and concentration levels for cytotoxicity and neurotoxicity. The mixture effects were predictable from the experimental single compounds’ concentration–response curves. These findings have important implications for the mixture risk assessment of PFAS.

## Abstract 2

Mixtures of 12 anionic PFAS at concentration ratios representative of their environmental occurrence revealed neurotoxicity and mitochondrial toxicity using *in vitro* assays, which follow the concentration addition model.

## Introduction

Per- and polyfluoroalkyl substances (PFAS) have been utilized in various products since the 1950s due to their effective water- and grease-repellent properties.^1,2^ Known for their persistence in the environment, PFAS have been detected in various matrices, including water, soil, plants, sludge, human and animal serum, and tissues.^3−8^ Legacy PFAS, such as perfluorooctanoic acid (PFOA) and perfluorooctane sulfonic acid (PFOS), have raised concerns regarding their impact on health and the environment. As a result, there has been an increased use of alternative PFAS, such as hexafluoropropylene oxide-dimer acid (HFPO-DA) and perfluorobutane sulfonic acid (PFBS), leading to their frequent occurrence in the environment.^9^

PFAS enter ecosystems through different pathways, including consumer goods, firefighting foams, industrial emissions, and effluents from wastewater treatment plants (WWTPs). Their solubility in water, mobility, and persistence contribute to the widespread contamination of the environment by PFAS.^10,11^ The incomplete removal of PFAS from wastewater and biosolids often results in the release of these substances into surface waters that receive WWTP effluents and in croplands where biosolids are applied.^12,13^

PFAS are structurally diverse and vary in chain lengths, molecular geometry, and head groups (e.g., carboxylates, sulfonates), which impacts their bioactivity and their tendency to bind with biomolecules.^14^ While there are over 10,000 PFAS^15^ listed in the chemical registry, very limited toxicity data are available, creating a significant gap in our understanding of their potential health effects.^2,16^ PFAS can adversely affect biological systems, especially the nervous system,^17^ through mechanisms such as oxidative stress,^18,19^ and receptor-mediated signaling pathways.^20,21^ Mixtures of PFAS have caused neurobehavioral and developmental toxicity in rats^22^ and altered epigenetic and transcriptomic regulations in mice.^23^ A mixture of persistent organic pollutants, including six PFAS at concentration ratios similar to those present in human blood, has been shown to affect neural connectivity *in vitro*.^24,25^ As summarized in a recent review by McCarthy et al.,^26^ few studies have investigated how PFAS act together in mixtures. Anionic PFAS mixtures mainly exhibited additive mixture effects on lipid metabolism in HepaRG cells.^27^

For risk assessment it is vital to know how chemicals act together in mixtures.^28^ Chemicals that act according to the same mode of action can be grouped in common assessment groups and their mixture effect typically follows the established mixture toxicity concept of concentration addition (CA).^29^ The model of independent action (IA) is typically applicable for mixtures of chemicals that have strictly different modes of action.^29^ While synergy and antagonism result from the interaction of mixture components, they are rare in realistic mixtures and most often caused by toxicokinetic interactions and not true toxicodynamic interferences.^30^

New approach methodologies (NAM) based on high-throughput screening (HTS) with *in vitro* cellular assays provide a way to screen molecular key events within adverse outcome pathways.^31,32^ Various NAM assays have been used to assess the effects of PFAS in general and specifically for developmental neurotoxicity.^33^ However, PFAS are challenging to test even in HTS assays. In a study focusing on 160 PFAS, only a limited number of PFAS tested showed activity in a developmental neurotoxicity HTS test battery, with the most anionic PFAS being inactive up to the highest concentrations tested.^33^ Anionic PFAS exhibited specific toxic effects unique to their chemical structure and interaction with biological targets such as the peroxisome-proliferator-activated receptor in cell line-based assays.^34^ Nonetheless, their activity in many *in vitro* assays can often be explained by nonspecific effects related to baseline toxicity associated with membrane disruption.^35^ Intracellular key events leading to neurodevelopmental disorders include synaptogenesis, degeneration of dopaminergic neurons, and disturbances of neuronal networks and their functions^31,36−38^ but also encompass cell death in neurons, mitochondrial dysfunction,^39^ activation of oxidative stress response, and endocrine disruption related to the thyroid hormone metabolism.^40^

**Figure 1:**
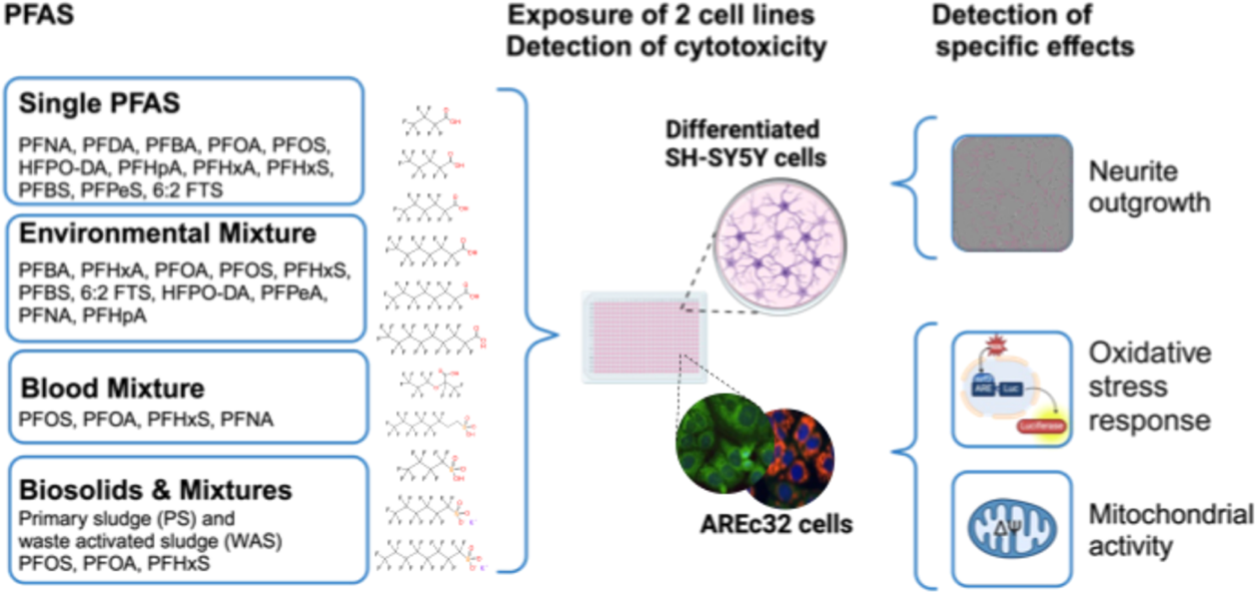
Study design. Differentiated SH-SY5Y and AREc32 cells were exposed to single PFAS and to several representative PFAS mixtures and extracts from the biosolids samples. Effects recorded after 24-h exposure included cytotoxicity in both cell lines. Inhibition of neurite length in the differentiated SH-SY5Y cells was detected by phase contrast imaging (top right– gray cell bodies, pink neurites). Oxidative stress response via the reporter gene activation of the Nrf2-ARE pathway as well as mitochondrial membrane potential inhibition was measured in AREc32 cells. Figure was partially created with BioRender.

In the present study, we evaluated mixture toxicity of PFAS at concentration ratios relevant in the environment and in human blood, focusing on their impacts on two cell lines (Figure 1). Human neuroblastoma (SH-SY5Y) cells differentiated into neuron cells were used as a screening tool to assess cytotoxicity and neurite outgrowth, serving as proxies for neurotoxicity.^38^ Oxidative stress response, mediated *via* the nuclear factor erythroid 2-related factor 2-Antioxidant Response Element (Nrf2-ARE) pathway, was quantified using the reporter protein luciferase, while mitochondrial toxicity was assessed using the mitochondrial membrane potential (MMP) indicator in the reporter gene cell line AREc32.^41^

We tested twelve anionic PFAS individually and in four realistic mixtures to evaluate how PFAS behave together (Figure 1). These twelve PFAS, identified by the United States (U.S.) Geological Survey from 2022,^42,43^ were selected for their distinct environmental relevance in WWTPs across the U.S. A four-component PFAS mixture, representing concentration ratios in human blood, was also designed based on mean blood concentrations from the U.S. National Health and Nutrition Examination Survey (NHANES).^44^ Furthermore, we extracted two types of biosolids from municipal WWTPs, quantified their PFAS content by liquid chromatography-tandem mass spectrometry(LC-MS/MS),^45^ and prepared representative mixtures in proportions of detected PFAS. Additionally, we compared the neurotoxic effects caused by the components of the biosolid extracts, which contained PFAS and other (unidentified) organic chemicals. This comparison aimed to estimate the contribution of PFAS to the complex mixture effects of organic chemicals in biosolids.

## Materials and Methods

### Mixture Preparation

Mixtures were prepared from methanolic stock solutions of 12 single PFAS (Table 1) at concentrations ranging from 0.037–0.186 M. For the PFAS mixture design, all the concentrations were converted from ng/g to molar (M) concentrations (Table 1). To calculate the molar fraction *p_i_* of each PFAS in the mixtures, eq 1 was used, where *C_i_* is the concentration of the component *i* and *C*_tot_ is the total concentration of all PFAS ().
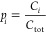
1

**Table 1:** PFAS Included in This Study, Design of the Environmental Mixture (Envmix), the Blood Mixture (Bloodmix), the Mixtures of Wastewater Activated Sludge (WASmix) and Primary Solid (PSmix)

		environmental mixture (envmix)					
chemical name	abbreviation	concentration *C_i_* (ng/L)	concentration *C_i_* in molar units (pM)	molar fraction *p_i_* in envmix	molar fraction *p_i_* in bloodmix^a^	molar fraction *p_i_* in WASmix^b^	molar fraction *p_i_* in PSmix^c^
perfluorobutanoic acid	PFBA	8.1	38.1	0.139			
perfluoropentanoic acid	PFPeA	6.1	23.1	0.086			
perfluorohexanoic acid	PFHxA	5.6	18.3	0.066	0.127	0.207	
perfluoroheptanoic acid	PFHpA	7.4	20.3	0.075			
perfluorooctanoic acid	PFOA	11.0	26.6	0.098	0.289	0.181	0.249
perfluorononanoic acid	PFNA	8.0	17.2	0.064	0.107		
perfluorobutane sulfonic acid	PFBS	4.9	16.3	0.061			
perfluoropentane sulfonic acid	PFPeS	5.1	13.7	0.051			
perfluorohexane sulfonic acid	PFHxS	5.9	14.7	0.055			
perfluorooctanoic sulfonic acid	PFOS	20	42.3	0.150	0.477	0.612	0.751
6:2 fluorotelomer sulfonic acid	6:2 FTS	10	23.4	0.086			
2,3,3,3-tetrafluoro-2-(heptafluoropropoxy)propanoic acid	HPFO-DA	5.8	17.6	0.065			

a Mean of detected concentrations in children’s serum: 96 μg/L (4.7 pM) PFOA, 0.81 μg/L (1.8 pM) PFNA, 0.83 μg/L (2.1 pM) PFHxS and 3.90 μg/L (7.8 pM) PFOS.

b Mean of detected concentrations in WAS: 4.2 ng/g_solid_ (10.1 pmol/g_solid_) PFOA, and 15.3 ng/g_solid_ (30.6 pmol/g_solid_) PFOS.^45^

c Mean of detected concentrations in PS: 8.5 ng/g_solid_ (20.7 pmol/g_solid_) PFHxA, 7.5 ng/g_solid_ (18.1 pmol/g_solid_) PFOA, and 30.6 ng/g_solid_ (61.2 pmol/g_solid_) PFOS.^45^

Mixtures were prepared by mixing methanolic stock solutions in appropriate fraction, aliquoting the desired quantity, evaporating the methanol, and reconstituting the final dosing solution in bioassay medium at 4× the highest concentration targeted.

### Mixture Design

Twelve PFAS in the environmental mixture (envmix) were selected based on high detection frequency observed in the U.S. WWTP effluents.^43^ The selected PFAS were mixed in the concentration ratios of the mean detected concentrations with fractions *p_i_* given in Table 1.

PFOA, PFNA, PFHxS, and PFOS were the most frequently detected PFAS in children’s serum, as reported in NHANES biomonitoring studies from 2013 to 2014.^44^ The blood mixture (bloodmix) was designed based on the geometric mean of serum concentrations for the U.S. population from the NHANES report with fractions *p_i_* in Table 1 according to the mean of the detected concentrations.

### WWTP Samples

Three grab samples of two types of biosolids, wastewater activated sludge (WAS) and lime-stabilized primary solids (PS), were collected from a WWTP. These samples were lyophilized, pulverized, and extracted as described by Dickman et al.^45^ The resulting extracts were concentrated, suspended in the starting mobile phase, and fortified with a ^13^C-labeled internal standard (MPFOA). The PFAS concentrations in the extracts were analyzed and previously reported by Dickman et al.^45^ Designed mixtures (PSmix and WASmix) were based on quantified amounts of PFHxA, PFOS and PFOA (Table 1).

Independently prepared extracts of these samples were dosed to the bioassays following previous procedures.^46^ The extracts had an enrichment factor (EF) of 250 g_solid_/L_methanol_. For dosing, an aliquot of the methanolic extract was blown down to dryness and then dissolved in bioassay medium at relative enrichment factors (REF) of up to 100 g_solid_/L_bioassay_.

### MitoOxTox Assay

The AREc32 cell line was used to test mitochondrial toxicity and oxidative stress response of individual PFAS, mixtures, and extracts as described by Lee et al.^41^ with details of the experiments given in the Supporting Information (SI), Text S2 and quality control measures described in Text S3.^47−49^ The effect concentration for 10% effect (EC_10_) or inhibitory concentration for 10% cytotoxicity (IC_10_) were derived from the concentration–response curves (CRC) as described in Text S4.^50^

### Neurotoxicity Assay

Differentiated human neuroblastoma SH-SY5Y cells were applied to test neurotoxicity of individual chemicals and mixtures according to Lee et al.^38^ with details of the experiments given in the SI, Text S5 and quality control measures in Text S6. The EC_10_ for shortening of neurite length (neurite outgrowth inhibition NOI) and IC_10_ for cytotoxicity were derived as above (Text S4).^50^

### Specificity Analysis

The ratio of IC_10_ to EC_10_ is a measure of the degree of specificity of effect, called the specificity ratio, SR (eq 2).
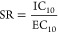
2

If SR > 10, the effect is highly specific. For 10 > SR > 1, the effect is valid but only moderately specific and could be caused indirectly by nonspecific toxicity that affects many different cellular processes. If the SR < 1, the inhibition of the neurite length is likely caused by nonspecific cytotoxicity, which kills the cells including the neurite, so the overall neurite length also decreases. In other words, only if the neurite length decreases at lower concentrations than those that cause cytotoxicity, the effect is specifically neurotoxic, else it is general toxicity.

Baseline toxicity, which is the minimum toxicity of every chemical, can be easily predicted from their tendency to accumulate in biological membranes, which can be simulated by the liposome–water distribution ratio *D*_lip/w_. Anionic PFAS have a slightly different baseline model than neutral PFAS because anionic chemicals bind stronger than neutral chemicals to proteins in bioassay medium.^51^ Therefore, there are separate baseline toxicity prediction models for anionic and neutral chemicals, which also differentiate between anionic and neutral PFAS.^35^Equation 3 is valid for cytotoxicity of anionic PFAS in the AREc32 cell line and eq 4 for cytotoxicity of anionic PFAS in SH-SY5Y cells.^35^ The *D*_lip/w_ of the anionic PFAS are either available in the literature^52,53^ or had been previously predicted,^35^ and are listed in Table 2.
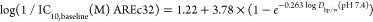
3
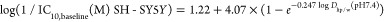
4

**Table 2:** Liposome–Water Distribution Ratio of the Anionic PFAS Species, *D*_lip/w_, and Cytotoxicity Inhibitory Concentrations IC_10_ for AREc32 and SH-SY5Y Cells and Effect Concentration EC_10_ for 10% Reduction of Neurite Length^a^

		**AREc32 cytotoxicity**			SH-SY5Y cytotoxicity			SH-SY5Y neurite outgrowth inhibition		
**PFAS**	**log*****D***_**lip/w**_**[*L***_**w**_**/*L***_**lip**_**]**	**IC**_**10**_	**SE IC**_**10**_	**TR**	**IC**_**10**_	**SE IC**_**10**_	**TR**	**EC**_**10**_	**SE EC**_**10**_	**SR**
PFBA	1.00^b^	3.92 × 10^–3^	5.02 × 10^–4^	2.06	1.95 × 10^–3^	8.86 × 10^–5^	3.97	2.13 × 10^–3^	1.20 × 10^–3^	0.92
PFPeA	1.75^d^	1.05 × 10^–3^	8.06 × 10^–5^	2.31	1.67 × 10^–3^	1.02 × 10^–4^	1.34	3.41 × 10^–3^	1.51 × 10^–3^	0.49
PFHxA	2.32^c^	2.82 × 10^–4^	1.76 × 10^–5^	4.02	1.23 × 10^–3^	1.13 × 10^–4^	0.82	1.23 × 10^–3^	1.72 × 10^–4^	0.99
PFHpA	2.91^c^	1.67 × 10^–4^	9.32 × 10^–6^	3.43	8.65 × 10^–4^	7.85 × 10^–5^	0.57	5.44 × 10^–4^	9.83 × 10^–5^	1.59
PFOA	3.52^c^	5.43 × 10^–5^	3.03 × 10^–6^	5.80	2.76 × 10^–4^	2.66 × 10^–5^	0.95	2.42 × 10^–4^	1.70 × 10^–5^	1.14
PFNA	4.25^c^	1.15 × 10^–4^	1.20 × 10^–5^	1.49	4.97 × 10^–4^	5.62 × 10^–5^	0.27	1.99 × 10^–4^	2.49 × 10^–5^	2.50
PFBS	3.51^c^	7.58 × 10^–4^	5.25 × 10^–5^	0.42	1.09 × 10^–3^	6.00 × 10^–5^	0.24	9.68 × 10^–4^	3.77 × 10^–5^	1.12
PFPeS	3.33^d^	2.82 × 10^–4^	2.08 × 10^–5^	1.33	4.92 × 10^–4^	2.36 × 10^–5^	0.64	5.72 × 10^–4^	8.76 × 10^–5^	0.86
PFHxS	4.13^c^	1.66 × 10^–4^	1.27 × 10^–5^	1.13	4.05 × 10^–4^	3.85 × 10^–5^	0.37	2.80 × 10^–4^	4.60 × 10^–5^	1.45
PFOS	4.89^c^	5.64 × 10^–4^	5.82 × 10^–5^	0.20	4.12 × 10^–4^	3.85 × 10^–5^	0.20	3.03 × 10^–4^	5.12 × 10^–5^	1.36
6:2 FTSA	3.87^d^	7.22 × 10^–4^	4.66 × 10^–5^	0.32	1.21 × 10^–2^	2.30 × 10^–3^	0.02	3.86 × 10^–3^	8.05 × 10^–4^	3.15
HFPO-DA	2.41^c^	4.22 × 10^–4^	2.65 × 10^–5^	2.40	1.18 × 10^–3^	5.58 × 10^–5^	0.76	2.80 × 10^–3^	5.61 × 10^–4^	0.42

a Full names of the abbreviated PFAS are given in Table 1. The toxic ratio TR is the ratio of the predicted IC_10_ of baseline toxicity and the measured IC_10_ (eq 5). The specificity ratio (SR) is the ratio of the predicted IC_10_ of baseline toxicity and the measured EC_10_ (eq 2).

b Experimental log *D*_lip/w_ from Droge.^52^

c Experimental log *D*_lip/w_ from Ebert et al.^53^

d Predicted log *D*_lip/w_ from Qin et al.^35^

The measured cytotoxicity IC_10_ can also be compared with baseline toxicity. The toxic ratio TR is a measure of the excess cytotoxicity (eq 5).
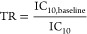
5

### Mixture Toxicity Evaluation

The 10% inhibitory concentration for cytotoxicity of a concentration-additive mixture IC_10_(CA) can be predicted with eq 6, if the n components *i*, present in fractions *p_i_*, with ∑*p_i_* = 1 act jointly according to CA.^29^
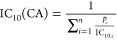
6

For low effect levels (<10%) and linear CRC (eq S1), the CA model simplifies to eq 7, which is equally valid for chemicals acting according to independent action (IA).^50^
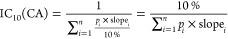
7

The same model can be applied for the effect concentration EC_10_(CA).

The slope of the CRC for the CA prediction (slope_CA_) is defined by eq 8 and its SE(slope _mixture_) by eq 9.^50^
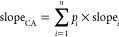
8
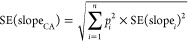
9

The IC_10_(CA) and EC_10_(CA) of the CA mixture prediction can then be derived by implementing the slope_CA_ and its SE into eqs S2 and S3. A measure of the quality of the mixture prediction is the index of prediction quality (IPQ),^54^ which is defined by eq 10.
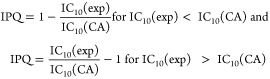
10

The contribution of one mixture component *i* to the overall mixture effect, Tox*_i_*, was calculated with eq 11.
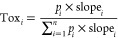
11

The relative effect potency, REP_i_, is the ratio between the EC_10_ of PFOA and that of chemical *i*.
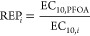
12

The CRC of the mixture is calculated by eq 13 for any effect level below 10%. Above 10% the predictions become nonlinear.^50^
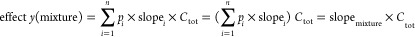
13

## Results and Discussion

### Measured Effects of Single PFAS

The assays were robust and repeatable as demonstrated by the quality control measures detailed in Text S3 (Figures S1 and S2) for the MitoOxTox and in Text S6 (Figure S3) for the neurotoxicity assay. In the MitoOxTox assay cytotoxicity was the dominant effect of the single PFAS (concentration–response curves, CRCs, in Figure S4, IC_10_ in Table 2). No activation of oxidative stress response was detected. MMP inhibition was detected only at concentrations that also caused cytotoxicity (Figure S4), which means that mitochondrial toxicity was a consequence of cytotoxicity and not a specific mode of action triggered by PFAS and no EC_10_ values could be derived.

All investigated PFAS caused cytotoxicity on differentiated SH-SY5Y cells (CRCs in Figure S5, IC_10_ in Table 2). The neurite outgrowth inhibition was often affected only at concentrations that caused cytotoxicity (Figure S5). Nevertheless, we recorded this end point and derived EC_10_ (Table 2) and included the end point of NOI in the mixture evaluation.

As there was a little difference in the two methods for quantification of confluency using phase contrast imaging with and without nuclei staining (Figure S6a), only the data using phase contrast imaging with nuclei staining will be reported below. The cytotoxicity IC_10_ (Table 2) agreed well between the two cell lines (Figure S6b) with the exception of 6:2 FTSA, which was less potent in SH-SY5Y.

### Comparison of Measured Cytotoxicity with Baseline Toxicity

**Figure 2:**
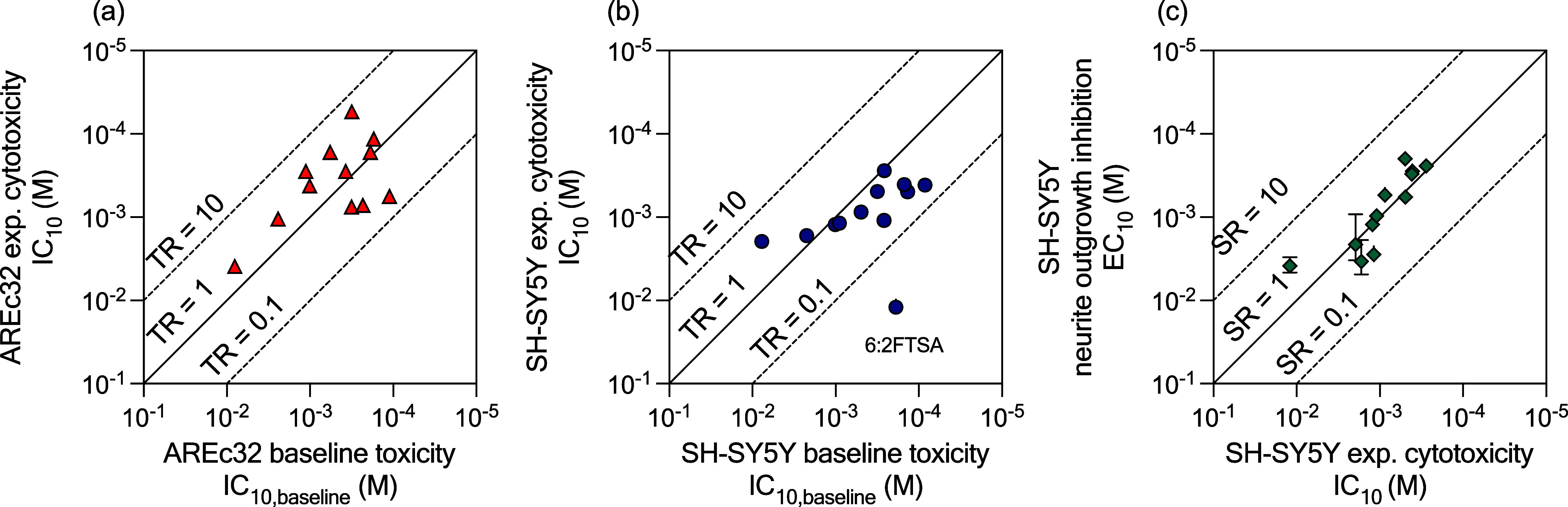
Comparison between predicted baseline toxicity IC_10,baseline_ and measured cytotoxicity IC_10_ for (a) AREc32 cells and (b) SH-SY5Y cells. (c) Comparison of measured cytotoxicity IC_10_ and neurite outgrowth inhibition EC_10_ in differentiated SH-SY5Y cells. TR, toxic ratio; SR, specificity ratio.

All PFAS in AREc32 (Figure 2a) and SH-SY5Y cells (Figure 2b) showed nonspecific cytotoxicity with a toxic ratio 0.1 < TR < 10 (Table 2). Only 6:2 FTSA had a TR of 0.02 in SH-SY5Y cells, which might be related to metabolism. The cytochrome P450 2D6 enzyme is constitutively expressed in differentiated SH-SY5Y cells,^55^ and other types are inducible.^56^ Because 6:2 FTSA is relatively degradable compared to all tested PFAS due to its ethane functional unit, it is likely that its low TR is caused by metabolism and formation of smaller perfluorinated carboxylic acids, which are less potent. This is also substantiated by AREc32 having a higher TR of 0.3 for 6:2 FTSA. AREc32 cells do not constitutively express cytochrome P450s; however, their expression can be induced as a response to exposure to xenobiotics.^57^ Therefore, it is reasonable that the TR is higher for SH-SY5Y cells, but still lower than 1.

The effects on neurite outgrowth inhibition occurred just around the experimental cytotoxicity with specificity ratio (SR) between 0.4 and 0.2 (Figure 2c, Table 2), which means that the effect was presumably a side effect of cytotoxicity and not a specific inhibition on neurite development. A more detailed analysis of the single chemicals effects and comparison with previous experiments^35^ is given in Text S7 and Figure S7.

### Mixtures

**Figure 3:**
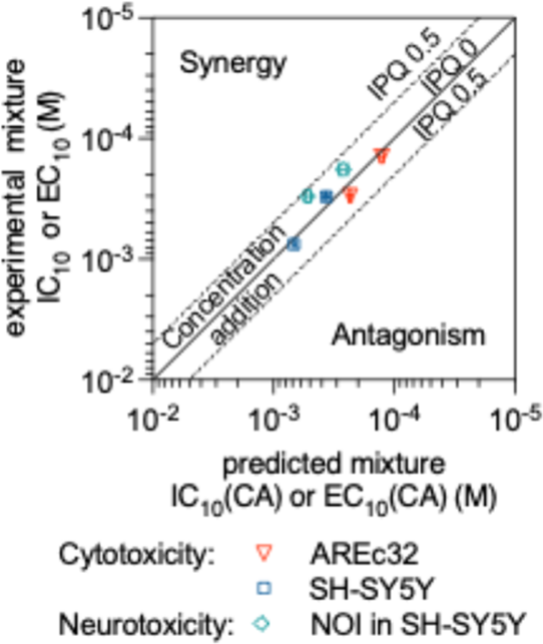
Comparison between the experimental mixture IC_10_ (inhibitory concentration causing 10% cytotoxicity) with the predicted mixture IC_10_(CA) calculated with the mixture model of concentration addition (CA) (eqs 6–9) for AREc32 and SH-SY5Y cells for the envmix and bloodmix; comparison of experimental and predicted EC_10_ (effect concentration causing 10% reduction of neurite length) for neurite outgrowth inhibition (NOI) in SH-SY5Y. The line corresponds to perfect agreement between model and prediction (index of prediction quality (eq 10) IPQ = 0), the dashed lines mark the area of IPQ up to 0.5. No data lay in the upper left corner, where synergistic effects would be displayed or the bottom right corner, where antagonistic effects would be displayed.

The mixtures, envmix and bloodmix, showed only cytotoxicity in the MitoOxTox assay (Figure S8) but the EC_10_ for neurite outgrowth inhibition could be derived in the neurotoxicity assay in addition to cytotoxicity IC_10_ (Figure S9, Table 3). Because all PFAS tested act as baseline toxicants, and mixture of baseline toxicants act according to CA,^58^ we can posit that the mixture effect follows CA. As we deduced the IC_10_ and EC_10_ from the linear portion of the CRC < 30% effect, the simplified CA model (eqs 8–10) was applied for mixture toxicity prediction. The resulting IC_10_(CA) and EC_10_(CA) are listed in Table 3 together with the IPQ (eq 10). Both designed mixtures envmix and bloodmix had an IPQ < 0.5, which confirmed that their mixture effect could be well predicted by CA for cytotoxicity in both cell lines and neurite outgrowth inhibition (Figure 3). The IPQs ranged from 0.11 to 0.28 for cytotoxicity (Table 3), which is an excellent agreement, and were slightly higher (0.32 and 0.42) for NOI but still within the prediction range for CA.

**Table 3:** Cytotoxicity Inhibitory Concentrations IC_10_ for AREc32 and SH SY5Y Cells and Effect Concentration EC_10_ for 10% Reduction of Neurite Length (NOI) for the Two Designed Mixtures Envmix and Bloodmix (Table 1)^a^

		**AREc32 cytotoxicity**			SH-SY5Y cytotoxicity			**SH-SY5Y neurite outgrowth inhibition**		
	**mixture**	**IC**_**10**_	**SE IC**_**10**_	**IPQ**	**IC**_**10**_	**SE IC**_**10**_	**IPQ**	**EC**_**10**_	**SE EC**_**10**_	**IPQ**
**envmix**	CA prediction	2.30 × 10^–4^	6.88 × 10^–6^		6.77 × 10^–4^	2.47 × 10^–5^		5.24 × 10^–4^	3.16 × 10^–5^	
	experimental	2.98 × 10^–4^	3.36 × 10^–5^	0.28	7.52 × 10^–4^	5.29 × 10^–5^	0.11	3.01 × 10^–4^	1.45 × 10^–5^	0.42
**bloodmix**	prediction	1.27 × 10^–4^	5.33 × 10^–6^		3.66 × 10^–4^	2.05 × 10^–5^		2.66 × 10^–4^	2.10 × 10^–5^	
	experimental	1.41 × 10^–4^	1.31 × 10^–5^	0.11	3.03 × 10^–4^	3.04 × 10^–5^	0.17	1.80 × 10^–4^	1.57 × 10^–5^	0.32

a The mixture IC_10_ and EC_10_ were predicted with the mixture model of concentration addition (CA, eqs 6–9), and the index of prediction quality (IPQ) was calculated with eq 10.

### Representative Environmental Mixture (Envmix)

**Figure 4:**
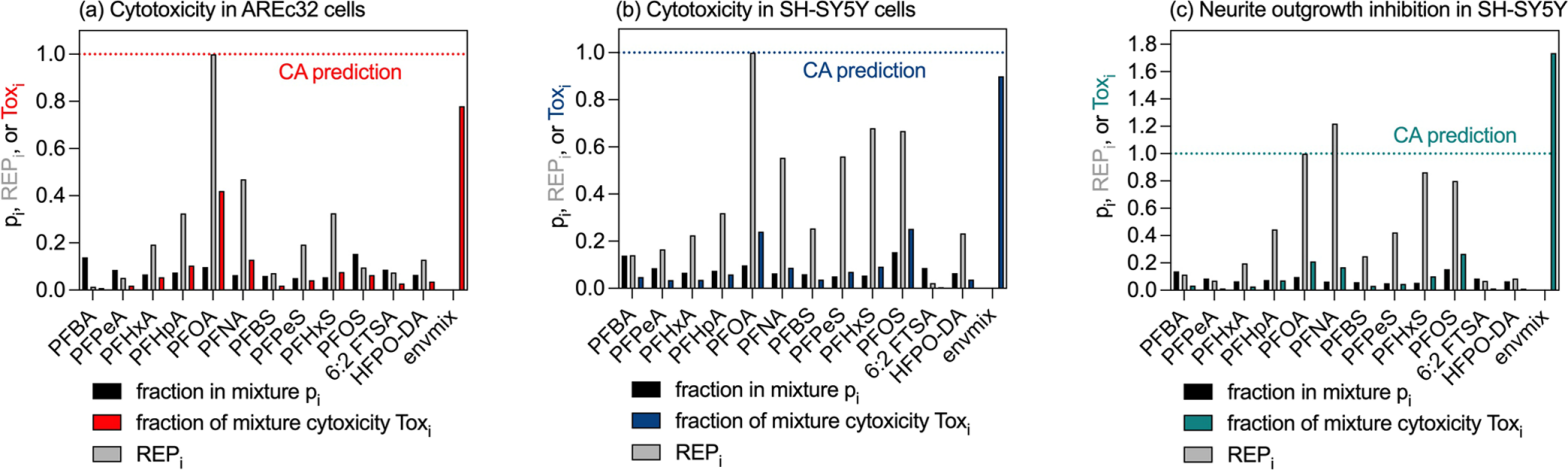
Environmental mixture (envmix): comparison of contribution of individual PFAS i to the fraction in the mixture (p_i_), their relative effect potency compared to PFOA (REP_i_ = IC_10,PFOA_/IC_10,i_ or EC_10,PFOA_/EC_10,i_) and their contribution to the mixture toxicity (Tox_i_, eq 13). (A) cytotoxicity in AREc32, (B) cytotoxicity in SH-SY5Y, (C) neurite outgrowth inhibition in SH-SY5Y.

The envmix contained 12 PFAS in relatively similar proportions (Table 1, Figures S8 and 4) and is representative of groundwater and surface water. The relative effect potency, REP_i_ in relation to PFOA (eq 12) is plotted as gray bars for all active bioassays in Figure 4. For easier visual comparison we plotted the fraction of effect (Tox_i_). The sum of the Tox_i_ of the CA prediction would be 1, and the experimental effect of the mixture was 0.78 for cytotoxicity in AREc32 (Figure 4a), 0.90 for cytotoxicity in SH-SY5Y (Figure 4b) and 1.74 for neurite outgrowth inhibition (Figure 4c), which means that the experiment came close to the prediction. Typically, any deviation up to a factor of 2 (0.5 < ΣTox_i_ < 2) can be considered as adequate prediction because this range is typically within the experimental variability of *in vitro* bioassays.

PFOA was by far the most cytotoxic of the 12 PFAS in the mixture. Despite its low concentration, it was the most important mixture effect driver for the cytotoxicity in AREc32 (Figure 4a). PFNA was the second most cytotoxic in AREc32 and despite its even lower concentration, it was the second most important contributor to the mixture effect. The mixture effect of 7 PFAS made up 90% of the mixture cytotoxicity. In order of contribution, these were PFOA (42%), PFNA (12.9%), PFHpA (10.4%), PFHxS (7.7%), PFOS (6.3%), PFHxA (5.4%), PFPeS (4.2%).

The cytotoxicity of envmix in SH-SY5Y cells was more balanced: 8 PFAS contributed to 90% of cytotoxicity because several additional PFAS had high REP_i_ (Figure 4b). The main mixture effect contributors were PFOS (25%), PFOA (24%), PFHxS (9.2%), PFNA (8.7%), PFPeS (7.0%), PFHpA (5.9%), PFBA (4.8%) and PFBS (3.7%).

With respect to neurite outgrowth inhibition, PFNA was more potent than PFOA and PFHxS, and PFOS was only slightly less potent than PFOA. Accordingly, PFOS dominated the mixture effect with a contribution (Tox_*i*_) of 26.6% despite a molar contribution (*p_i_*) of 15%, followed by PFOA (21.2%), PFNA (16.6%), PFHxS (10.3%), PFHpA (7.2%), PFPeS (4.7%) and PFBA (3.4%) (Figure 4c).

### Representative Blood Mixture (Bloodmix)

**Figure 5:**
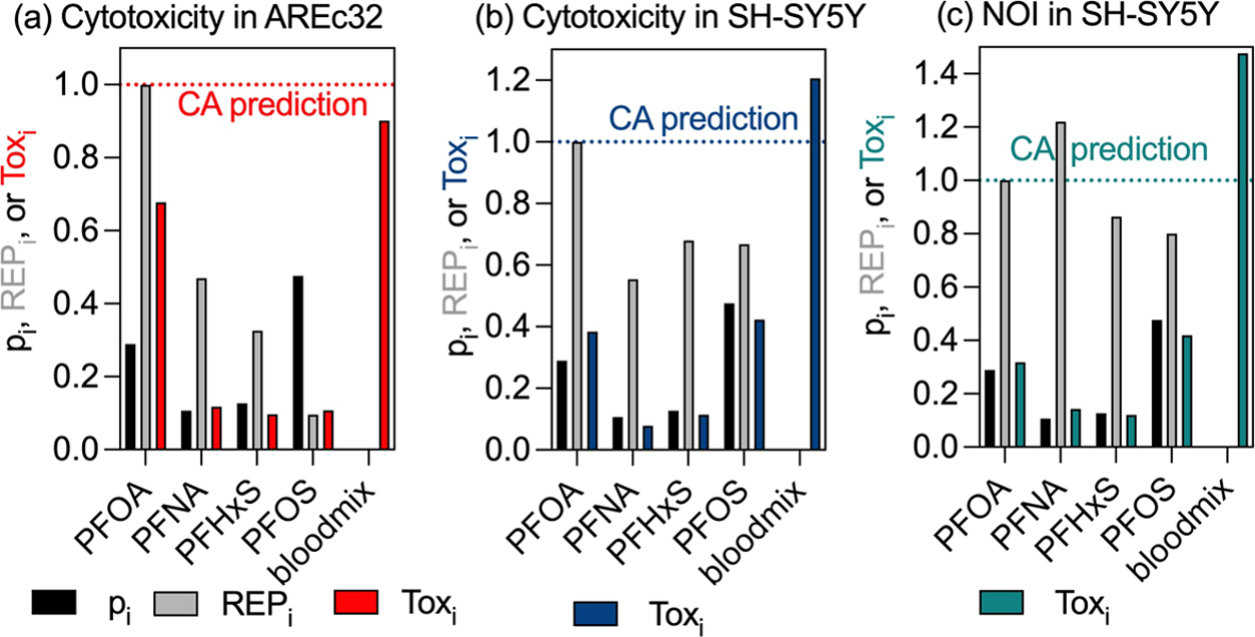
Blood mixture (bloodmix): comparison of contribution of individual PFAS to the fraction in the mixture (p_i_), their relative effect potency compared to PFOA (REP_i_ = IC_10,PFOA_/IC_10,i_ or EC_10,PFOA_/EC_10,i_) and their contribution to the mixture toxicity (Tox_i_, eq 13). (A) cytotoxicity in AREc32, (B) cytotoxicity in SH-SY5Y, (C) neurite outgrowth inhibition (NOI) in SH-SY5Y.

The bloodmix had only 4 components. PFOA dominated the cytotoxicity in both cell lines (Figure 5a,b). Despite its molar contribution being only 29%, it triggered 68% of the cytotoxicity in AREc32 (Figure 5a) and 38% in SH-SY5Y (Figure 5b). Neurite outgrowth inhibition was almost equally attributed to PFOA (38%) and PFOS (43%). PFNA had only a low molar fraction (10%) but the highest REP_i_ of the four mixture components, resulting in 14% contribution to the mixture effect (Figure 5c).

### How to Communicate Mixture Effects?

The calculations used for the mixture effect predictions are not too complex given that we worked in the linear range of the CRCs, where effects and concentrations scale linearly. Nevertheless, the Tox_i_ descriptors are not intuitive. We can use the analogy of the “risk cup” that has been recently phrased for mixture risk assessment,^59^ where all components of a mixture are translated into a common currency and added up. We can use bioanalytical equivalent concentrations (BEQ_chem_) to translate the contribution of any mixture component *i* as the concentration that an equivalent quantity of a reference compound would have. Here, we use PFOA as reference chemical and express effects as PFOA equivalent concentration PFOA-EQ_chem_. PFOA-EQ*_i_* for each mixture component *i* can be computed from the REP*_i_,* and its concentration, *C*_*i*_ (eq 14).^60^ The mixture effects PFOA-EQ_chem_ are the sum of individual PFOA-EQ*_i_.*
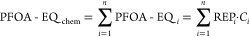
14

This calculation is only made possible once we have established that the mixture of anionic PFAS could be predicted by CA for all investigated end points, mixture compositions, and ratios. PFOA-EQ can also be expressed in units of ng/L for a more intuitive comparison with analytically determined concentrations because it is convention in the field of analytical chemistry to use mass-based concentrations. However, toxicology is based on the action of molecules, therefore molar concentrations are preferred in environmental toxicology for the mixture calculations (REP_i_ are molar ratios) but the PFOA-EQ are at the end converted back to ng/L for easier communication of results. It must be noted that PFOA-EQ does not mean that the same amount PFOA is in the mixture, but that the mixture will have the same effect as if such a concentration of PFOA were present.

The concentration of PFOA was 11 ng/L in the envmix.^43^ Taking the mixture effect of the additional 11 anionic PFAS into account, the predicted PFOA-EQ_chem_ were 26 ng/L for cytotoxicity in AREc32, 46 ng/L for cytotoxicity in SH-SY5Y and 52 ng/L for NOI (Table S2). The PFOA-EQ_chem_ differ for each end point due to variations in REP_i_ of the mixture components (Table S2).

The bloodmix, which was based on NHANES biomonitoring data, comprised only of four components, and while it contained only 2.0 ng/L PFOA, the PFOA-EQ_chem_ were 2.9 ng/L for cytotoxicity in AREc32, 5.1 ng/L for cytotoxicity in SH-SY5Y and 6.2 ng/L for NOI (Table S2). We can also calculate the PFOA-EQ_bio,mix_ directly from the experimental effect data of the designed mixtures (eq 15). PFOA-EQ_bio,mix_ and PFOA-EQ_chem_ agreed (Table S2) as expected for CA. The ratios of PFOA-EQ_bio,mix_ to PFOA-EQ_chem_ varied from 0.78 to 1.74 ng/L for the envmix and 0.90 to 1.48 for the bloodmix (Table S2), which is equivalent to the Tox_i_ in Figures 4 and 5.
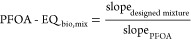
15

### Effects of Biosolid Extracts

The CRCs of the extracts of PS and WAS indicated activity in all end points in the MitoOxTox assay (Figure S10) and the neurotoxicity assay (Figure S11). The extracts even activated the oxidative stress response and inhibited the MMP, which were not activated/inhibited by PFAS individually or by the designed mixtures PSmix and WASmix. Evidently, there are many more chemicals in the biosolid extracts beyond PFAS that can trigger these specific effects. The three independent measurements using the extract of the same biosolid sample had variable IC_10_ and EC_10_ (Table S1), which is presumably caused by heterogeneities of the biosolid. No blanks could be obtained, so further investigation was not possible.

The designed mixtures of PSmix and WASmix were active in MMP (Figure S12) and NOI (Figure S13) and showed cytotoxicity in both cell lines but did not activate oxidative stress response just like the mixture components. Although only two and three PFAS were detected in PS and WAS and were included in the designed mixtures PSmix and WASmix, we performed the same mixture diagnostic analysis as for envmix and bloodmix. The IPQ were within the validity range for CA (IPQ < 0.5) for the NOI, but cytotoxicity had a tendency toward antagonism (Figure S14). In PSmix, PFOA dominated cytotoxicity in AREc32 and PFOS dominated cytotoxicity and NOI in SH-SY5Y cells (Figure S15). Potency differences between the three components of WAS (PFOA, PFHxS, PFOS) were small in the neurotoxicity assays and accordingly all components contributed to the mixture effect, while cytotoxicity in AREc32 cells was dominated by PFOA (Figure S16). The PFOA-EQ_bio,mix_ of the designed mixtures and the predicted PFOA-EQ_chem_ agreed within a factor of 5 (Table 4).

**Table 4:** Effects of Mixture Components Expressed as PFOA Equivalent Concentrations (PFOA-EQ) of the Experimental Mixture Effect (PFOA-EQ_bio_) of Primary Solid (PSmix) and Wastewater Activated Sludge (WASmix) and their Experimental (PFOA-EQ_bio,mix_) and Predicted (PFOA-EQ_chem_) Mixture Effect of the Two (PSmix) or Three (WASmix) PFAS Detected and Quantified in the Samples

	PSmix			WASmix		
abbreviation	PFOA-EQ_chem,i_ (ng_PFOA_/g_solid_) or (mg_PFOA_/g_solid_) cytotoxicity AREc32	PFOA-EQ_chem,i_ (ng_PFOA_/g_solid_) or (mg_PFOA_/g_solid_) cytotoxicity SH SY5Y	PFOA-EQ_chem,i_ (ng_PFOA_/g_solid_) or (mg_PFOA_/g_solid_) NOI	PFOA-EQ_chem,i_ (ng_PFOA_/g_solid_) or (mg_PFOA_/g_solid_) cytotoxicity AREc32	PFOA-EQ_chem,i_ (ng/g) or (mg/g) cytotoxicity SH SY5Y	PFOA-EQ_chem,i_ (ng/g) or (mg/g) NOI
	4.20	4.20	4.20	7.50	7.50	7.50
PFOA-EQ_i_ of PFHxS (ng_PFOA_/g_solid_)				2.8	5.82	3.17
PFOA-EQ_i_ of PFOS (ng_PFOA_/g_solid_)	1.22	8.45	10.1	2.44	16.9	8.42
PFOA-EQ_chem_ (ng_PFOA_/g_solid_)	5.42	12.6	14.3	12.73	30.3	10.1
PFOA-EQ_bio, mix_ (ng_PFOA_/g_solid_) designed mixture	1.09	4.91	9.16	3.15	17.7	20.4
PFOA-EQ_bio_ (mg_PFOA_/g_solid_) extract	5.80	32.2	88.3	3.60	36.6	50.3
fraction of effect in extract explained by PFAS	9.34 × 10^–7^	3.91 × 10^–7^	1.62 × 10^–7^	3.54 × 10^–6^	8.27 × 10^–7^	2.01 × 10^–7^

More interestingly, we observed that PFOA-EQ_bio,mix_ of the designed mixtures were 10^6^ times lower than the PFOA-EQ_bio_ of the entire extract (Table 4). PFOA-EQ_bio_ can be directly derived for the extracts of the PS and WAS samples from their IC_10_ and EC_10_ with eq 16.
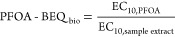
16

It should be noted that there are many more PFAS and other chemicals in biosolids that may have contributed to the toxicity in the extracts. However, because of the high persistence of PFAS, it is likely that PFAS concentrations in environments where biosolids are applied are more important relative to the other biodegradable chemicals that also contribute to biosolids’ toxicity.

### Implications for the Risk Assessment of PFAS

The comparison between PFOA concentration and PFOA-EQ_chem_ of the designed mixtures clearly demonstrates that replacing one PFAS by another will hardly mitigate risks posed by PFAS. PFOA-EQ_chem_ is a simple measure of the mixture effects and for any additional PFAS we add to the mixture that is bioactive, the PFOA-EQ_chem_ will inevitably increase. A recent study used cytotoxicity in HepG2 cells to investigate 50 complex mixtures that contained PFOA, PFNA and PFHxS among other organic chemicals and metals.^61^ Only 6 of 50 components had slightly antagonistic effects, most acted according to CA. The results of our study on PFAS mixture toxicity are reasonable considering that interactive mixture effects are more common in mixtures of metals and organics.^62^

It has been proposed that the relative potency factor approach can be used for the mixture risk assessment of PFAS.^63^ Bil et al.^64^ demonstrated the utility of this approach on a case study of liver toxicity (weight gain) on male rats that were orally dosed with PFAS for 42 to 90 days. They derived relative potency factors for this end point that ranged from 0.001 to 10, while the REP for cytotoxicity ranged from 0.01 to 1 but relative ranges agreed well (Figure S17). It should be checked if cytotoxicity to a liver cell line gives even better associations between relative potencies *in vivo* and *in vitro*. The relative potency factor approach in risk assessment implies concentration-additive mixture effects. The validity of the assumption of concentration addition is hardly ever tested *in vivo* because such experiments are expensive. The present *in vitro* study helps to justify this mixture toxicity assumption.

Most importantly, we demonstrated that all tested anionic PFAS were toxic to neurons at concentrations close to where nonspecific baseline toxicity occurs. As baseline toxicity is predictable from the physicochemical descriptor *D*_lip/w_,^35^ and concentration-additive mixture effects at low effect levels follow a simple prediction model,^60^ it is possible to predict the mixture effects of PFAS with high confidence. Colnot et al.^65^ have proposed to separate perfluorocarboxylic and perfluoro-sulfonic acids in independent assessment groups for risk assessment but the present study does not support this separation because all mixture effects were consistent with CA and hence should be grouped into a common assessment group for risk assessment.

However, one limitation of the present study is that only anionic PFAS were combined in mixtures. Future work should go beyond these homogeneous groups of perfluorocarboxylic and perfluorosulfonic acids and should include neutral PFAS, and other polyfluorinated chemicals. Extension to other, especially specific, end points and inclusion of other organic chemicals are the natural next step, but the present work lays the foundation for a new approach on how to tackle the risks of PFAS mixtures in various environmental matrices.

## Supporting Information Available

Additional information on chemicals, experimental details, concentration–response curves, additional analyses (PDF)

## Abbreviations

CA: concentration addition
CRC: concentration–response curves
EC_10_: effect concentration for 10% effect
PFAS: per- and polyfluoroalkyl substances
HTS: high-throughput screening
IA: independent action
MMP: mitochondrial membrane potential
NAM: new approach methodologies
NOI: neurite outgrowth inhibition
PS: primary solid
(R)EF: (relative) enrichment factor
SR: specificity ratio
TR: toxic ratio
WAS: wastewater activated sludge
WWTP: wastewater treatment plants
