# Supplementary material for: Neurotoxic Effects of Mixtures of Perfluoroalkyl Substances (PFAS) at Environmental and Human Blood Concentrations
Source: Environ Sci Technol. 2024 Sep 11;58(38):16774–84. doi: 10.1021/acs.est.4c06017 (PMC11428134; doi:10.1021/acs.est.4c06017)
Supplement: Supplementary file 1 — es4c06017_si_001.pdf [file es4c06017_si_001.pdf]

Supporting Information

Neurotoxic Effects of Mixtures of Perfluoroalkyl Substances (PFAS) at Environmental and Human Blood Concentrations

Karla Ríos-Bonilla†, Diana S. Aga†, Jungeun Lee‡, Maria König‡, Weiping Qin‡, Judith R. Cristobal†, Gunes Ekin Atilla-Gokcumen† and Beate I. Escher‡*

†Department of Chemistry, University at Buffalo - The State University of New York, Buffalo, NY 14260, USA

‡ Department of Cell Toxicology, Helmholtz-Centre for Environmental Research – UFZ, Leipzig 04318, Germany

Number of pages: 34

Number of figures: 14

Number of Tables: 2

Table of Content

Supplementary texts

Text S1. Materials

Text S2. MitoOxTox assay

Text S3. Quality control of the MitoOxTox assay

Text S4. Data evaluation

Text S5. Neurotoxicity assay

Text S6. Quality control of the neurotoxicity assay

Text S7. Detailed discussion of the baseline toxicity analysis and comparison with literature data

Figures

Figure S1. Concentration-response curves of the positive control t-butylhydroquinone for oxidative stress response in the MitoOxTox assay.

Figure S2. Concentration-response curves of the positive control azoxystrobin for inhibition of the mitochondrial membrane potential (MMP) in the MitoOxTox assay.

Figure S3. Concentration-response curves of the positive control narciclasine in the neurotoxicity assay.

Figure S4. Concentration-response curves of single PFAS in the MitoOxTox assay.

Figure S5. Concentration-response curves of single PFAS in the neurotoxicity assay.

Figure S6 (a) Comparison of the cytotoxicity IC_{10} of AREc32 cells quantified with phase contrast imaging with and without nuclei staining. (b) Comparison of the cytotoxicity IC_{10} between the SH-S5Y5 and AREc32 cells.

Figure S7. Measured inhibitory concentration causing 10% cytotoxicity IC_{10} of single chemicals for (a) AREc32 cells and (b) SH-SY5Y cells plotted against the hydrophobicity expressed as $logD_{lip/w}$ and comparison with the baseline toxicity QSARs for anionic PFAS

Figure S8. Top left: the pie chart represents the fraction p_i of the 12 PFAS (i) present in the mixture. Concentration-response curves of mixtures in the MitoOxTox assay.

Figure S9. Concentration-response curves of mixture in the neurotoxicity assay: (A) envmix (B) bloodmix.

Figure S10. Concentration-response curves of extracts from (A-C) wastewater (WAS) and (D-E) primary biosolids (PS) in the MitoOxTox assay.

Figure S11. Concentration-response curves of extracts from (A-C) wastewater activated sludge (WAS) and (D-E) primary biosolids (PS) in the neurotoxicity assay.

Figure S12. Pie chart represents the fraction p_i of the two or three PFAS (i) present in the mixture. Concentration-response curves of designed wastewater mixtures in the MitoOxTox assay. (A) WASmix, (B) PSmix.

Figure S13. Pie chart represents the fraction p_i of the two or three PFAS (i) present in the mixture. Concentration-response curves of designed wastewater mixtures in the neurotoxicity assay. (A) WASmix, (B) PSmix.

Figure S14. Comparison of in vitro REP of cytotoxicity and relative potency factors *in vivo* for liver toxicity (weight gain) on male rats that were orally dosed for 42 to 90 days.

Tables

Table S1. Effect concentrations $EC_{IR1.5}$ for activation of oxidative stress response, EC_{10} for inhibition of the mitochondrial membrane potential (MMP), cytotoxicity inhibitory

concentrations IC_{10} for AREc32 and SH-SY5Y cells and effect concentration EC_{10} for 10% reduction of neurite length by the sample extracts.

Table S2. Effects of mixture components expressed environmental mixture (envmix), the blood mixture (blood mix), the mixtures of wastewater activated sludge (WASmix) and primary solid (PSmix).

Text S1. Materials

The following PFAS standards were purchased from Millipore Sigma®: perfluorobutanoic acid (PFBA), perfluorobutane sulfonic acid (PFBS), perfluorooctanoic acid (PFOA), perfluorooctane sulfonic acid (PFOS), perfluorodecanoic acid (PFDA), and perfluorodecane acid (PFDA), perfluorohexane sulfonate (PFHxS), perfluorohexanoic acid (PFHxA), hexafluoropropylene oxide-dimer acid (HFPO-DA), perfluoropentanoic acid (PFPeA), perfluoroheptanoic acid (PFHpA), perfluoropentane sulfonic acid (PFPeS). Isotopically-labelled standard (13C-labelled) PFOA (MPFOA) was purchased from Wellington Laboratories (Overland, KS). The automated liquid-handling platform Hamilton Microlab STAR was obtained from Hamilton Star, Bonaduz, Switzerland. Resuspension of cells was transferred using a Tecan D300e Digital Dispenser, which was acquired from Tecan, Crailsheim, Germany. Nuclear Green LCS1 and propidium iodide were purchased from Sigma Aldrich. Sep-pak™ C18 solid phase extraction (SPE) cartridges (6cc, 500 mg) were purchased from Waters (Milford, MA). Liquid chromatography–mass spectrometry (LC-MS) grade acetonitrile and methanol for instrumental analysis and for extraction solvent were obtained from Omnisolv® through Millipore Sigma (Saint Louis, MO) and Fisher Chemical (Pittsburg, PA), respectively. The American Chemical Society (ACS) grade glacial acetic acid and ammonium acetate were purchased from J. T. Baker (Philipsburg, NJ).

Text S2. MitoOxTox assay

Experimental details of the MitoOxTox assays were already described by Lee et al.[1] Assay medium contained 90% of DMEM (Gibco, 31966-021), 10% of fetal bovine serum (Gibco, 10099-141) and 100 U/mL penicillin and 100 µg/mL streptomycin (Gibco, 15140-122). The cells were plated at density of 10,000 cells/well in a white wall/clear bottom 384-well plate (Greiner, 781944) and incubated for 24 h. Chemical/samples were treated to the cells and the plate was further incubated for further 24 h. After the exposure, imaging was performed to evaluate cytotoxicity and inhibition in mitochondrial membrane potential (MMP). The blue-fluorescent nuclei stain Hoechst 33342 (Invitrogen, H3570) and the mitochondrial membrane potential indicator (m-MPI; Codex BioSolutions, CB-80600) were added to the cell plate and the plate was incubated for 30 mins. Phase-contrast and fluorescence images were acquired with an ImageXpress High-Content Imaging System (Molecular Devices, Sunnyvale, CA, USA) with a 10X objective lens. Images were analyzed using deep learning-based cellular segmentation method CellPose[2] and an open-source software CellProfiler (ver 4.2.5).[3] Cell confluence indicated by the phase-contrast images and the blue-stained nuclei was used as a measure of cell viability and red/green fluorescence ratio of MMP was the measure of mitochondrial toxicity.[4] Cell confluence was also quantified with the IncuCyte® S3 live cell imaging system as for the SH-SY5Y cells described above. After image acquisition, oxidative stress response was measured by quantifying the reporter protein luciferase. The cells were washed with PBS, lysed with lysis buffer and treated with substrate buffer containing D-luciferin (AAT Bioquest, ABD-12506). A Tecan Infinite® M1000 plate reader was used to measure luminescence. Experiments were repeated three times independently and evaluated with a common concentration-response model.

Text S3. Quality control of the MitoOxTox assay

The positive control for the endpoint of oxidative stress response is *tert*-butylhydroquinone (*t*BHQ). *t*BHQ activated the reporter gene with an $EC_{IR1.5}$ of 2.40±0.07 µM (Figure S1), which is in the same range as the long-term record in our laboratory and agrees with previous $EC_{IR1.5}$ of 3.14 µM in the MitoOxTox assay[1] and 1.32 µM in Escher et al.[5] No cytotoxicity was observed for *t*BHQ (Figure S1). No inhibition of the mitochondrial membrane potential (MMP) was observed but the signal increased (= negative inhibition) and no EC_{10} could be derived (Figure S1).

The positive control for the endpoint of mitochondrial toxicity is azoxystrobin (Figure S2). Azoxystrobin had an EC_{10} of 37.0 ± 7.3 µM for inhibition of MMP, similar to the previously reported EC_{10} of 15.9 µM.[1] Azoxystrobin was not cytotoxic up to the highest tested concentration of 67 µM and activated the oxidative stress with an $EC_{IR1.5}$ of 85.2 ± 6.7 µM.

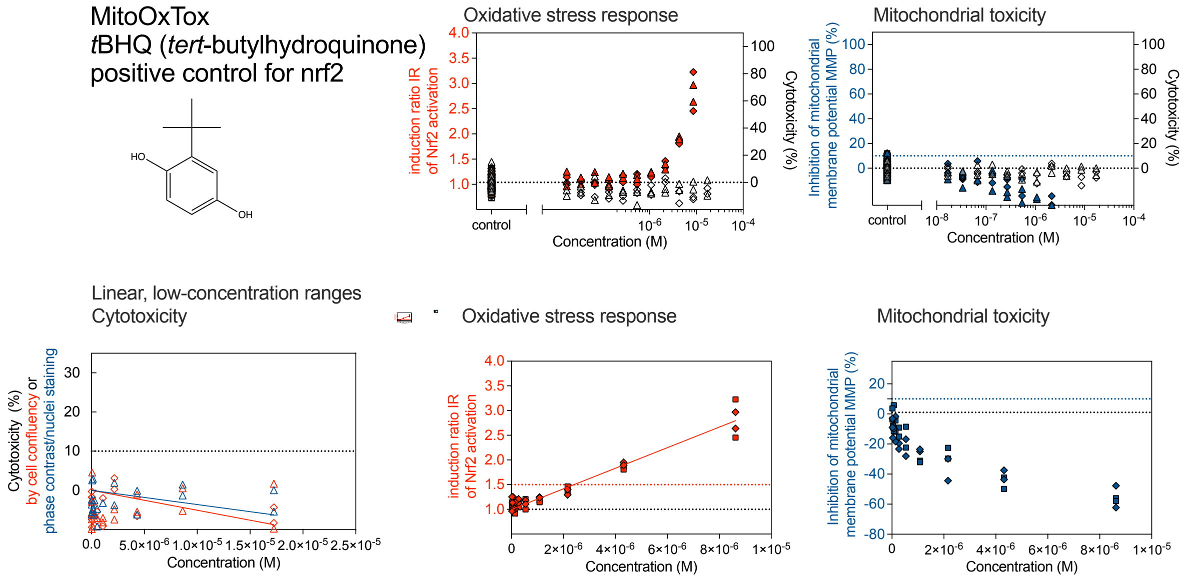

Figure S1. Concentration-response curves of the positive control t-butylhydroquinone for oxidative stress response in the MitoOxTox assay. The top row depicts the CRCs for the oxidative stress response (activation of the Nrf2-ARE adaptive stress response pathway) and the mitochondrial toxicity (inhibition of the mitochondrial membrane potential MMP) on a logarithmic concentration scale to visualize all datapoints in relation to cytotoxicity. The bottom row only includes valid data on a linear concentration scale and includes the fitted linear CRCs for cytotoxicity (left), oxidative stress response (middle) and mitochondrial toxicity (right). Different symbols represent independent experiments.

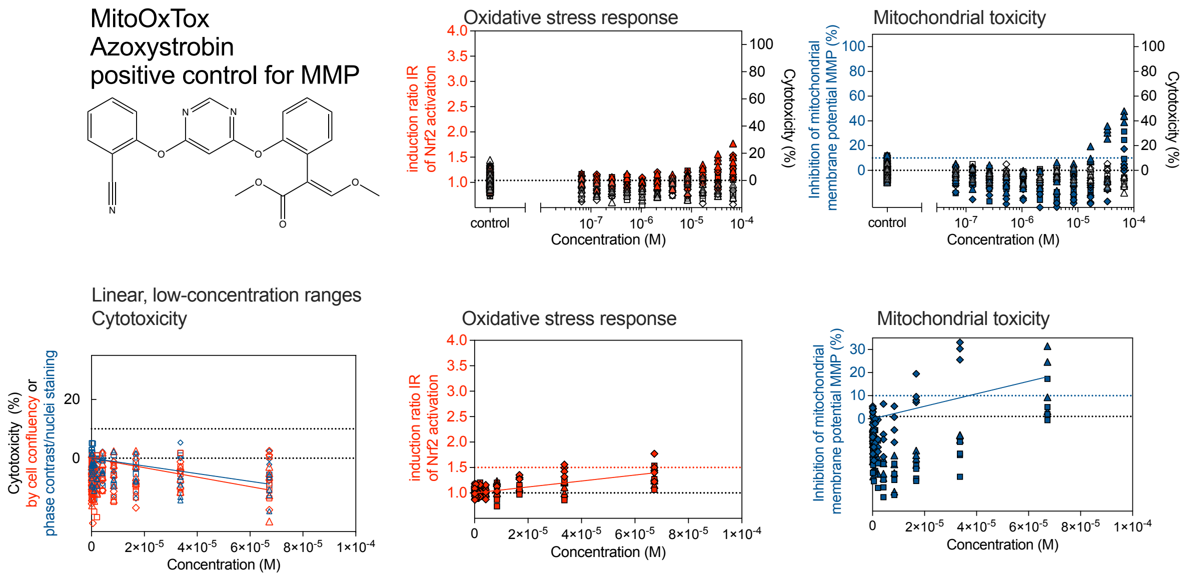

Figure S2. Concentration-response curves (CRC) of the positive control azoxystrobin for inhibition of the mitochondrial membrane potential (MMP) in the MitoOxTox assay. The top row depicts the CRCs for the oxidative stress response (activation of the Nrf2-ARE adaptive stress response pathway) and the mitochondrial toxicity on a logarithmic concentration scale to visualize all datapoints. The bottom row only includes valid data on a linear concentration scale and includes the fitted linear CRCs for cytotoxicity (left), oxidative stress response (middle) and mitochondrial toxicity (right). Different symbols represent independent experiments.

Text S4. Data evaluation

Concentration-response curves (CRCs) are often fitted by log-logistic or Hill models. However, realistic mixtures occur at very low concentrations with low effect levels <30% effect, where most monotonic CRCs of *in vitro* assays simplify to a line with an intercept zero (eq 2).[6]

$$\text{Effect } y_i = \text{slope}_i \times C_i \qquad \text{(S1)}$$

The effect concentration for 10% effect (EC_{10}) or 10% cytotoxicity (IC_{10}) and its standard error SE can then be calculated with eq 3 and 4.[6]

$$EC_{10} = \frac{10\%}{\text{slope}} \qquad \text{(S2)}$$

$$SE(EC_{10}) \approx \frac{10\%}{\text{slope}^2} \times SE(\text{slope}) \qquad \text{(S3)}$$

Only concentrations C_i < IC_{10} of cytotoxicity should be used for the evaluation of specific effects, e.g., the inhibition of neurite outgrowth or the inhibition of the mitochondrial membrane potential because if specific effects occur at cytotoxic concentrations, they are probably only indirect consequences of cytotoxicity and not causative of toxicity. However, we relaxed this C_i < IC_{10} criterion, because even PFAS that showed no effects on their own because they were masked by cytotoxicity can still contribute to mixture effects at lower non-cytotoxic concentrations. Therefore, we derived EC_{10} even if they were higher than IC_{10}, which are strictly no valid effects.

Text S5. Neurotoxicity assay

Details of the neurotoxicity assay are given in literature.[7] The cells were differentiated with 10 µM all-trans retinoic acid (Sigma-Aldrich, R2625) for 72 h in flasks and seeded at density of 3,100 cells/well in Collagen I-coated 384-well plates (Corning, 354667). The assay medium was composed of phenol-red free Neurobasal™ medium (Gibco, 12348017) supplemented with 2% B-27™ Supplement (Gibco, 17504044), 2 mM GlutaMAX™ (Gibco, 35050061), and 100 U/mL penicillin and 100 µg/mL streptomycin. The cell plates were incubated for 24 h and treated with chemical/samples for further 24 h.

At the end of exposure, phase-contrast image was obtained and analyzed by an IncuCyte® S3 live cell imaging system (Essen BioScience, Ann Arbor, Michigan, USA) to measure neurite length. Then, the cells were stained with Nuclear Green™ LCS1 (Abcam, ab138904) and propidium iodide (Sigma-Aldrich, 81845) for 1.5 h to obtain fluorescence images. The number of total and dead cells was quantified based on nuclei objects, identified from fluorescence images using IncuCyte® S3 software.

Text S6. Quality control of the neurotoxicity assay

The positive control for the neurotoxicity assay is narciclasine (Figure S3). Narciclasine had an EC_{10} of 6.6 ± 0.4 nM for inhibition of neurite outgrowth, similar to the previously reported EC_{10} of 3.9 ± 0.4 nM.[7] Cytotoxicity occurred at much higher concentrations with an IC_{10} of 22.9 ± 12.1 µM, which is consistent with previous studies (IC_{10} 12.1 µM).[7] The specificity ratio SR was 35, consistent with literature of 42.[7]

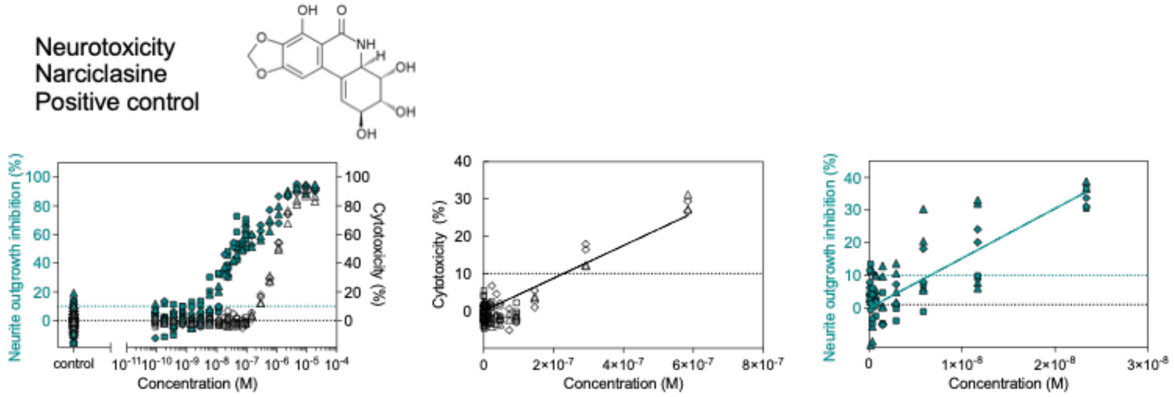

Figure S3. Concentration-response curves (CRC) of the positive control narciclasine in the neurotoxicity assay. The left curve depicts the CRCs for the inhibition of neurite outgrowth (NOI) and cytotoxicity on a logarithmic concentration scale to visualize all datapoints. The middle plot only includes the linear range of the cytotoxicity CRC (<30% cytotoxicity) on a linear concentration scale and the line corresponds to the fitted linear CRCs for cytotoxicity. The right plot only includes the linear range of the NOI CRC (<30% NOI, non-cytotoxic) on a linear concentration scale and the line corresponds to the fitted linear CRC for NOI. Different symbols represent independent experiments.

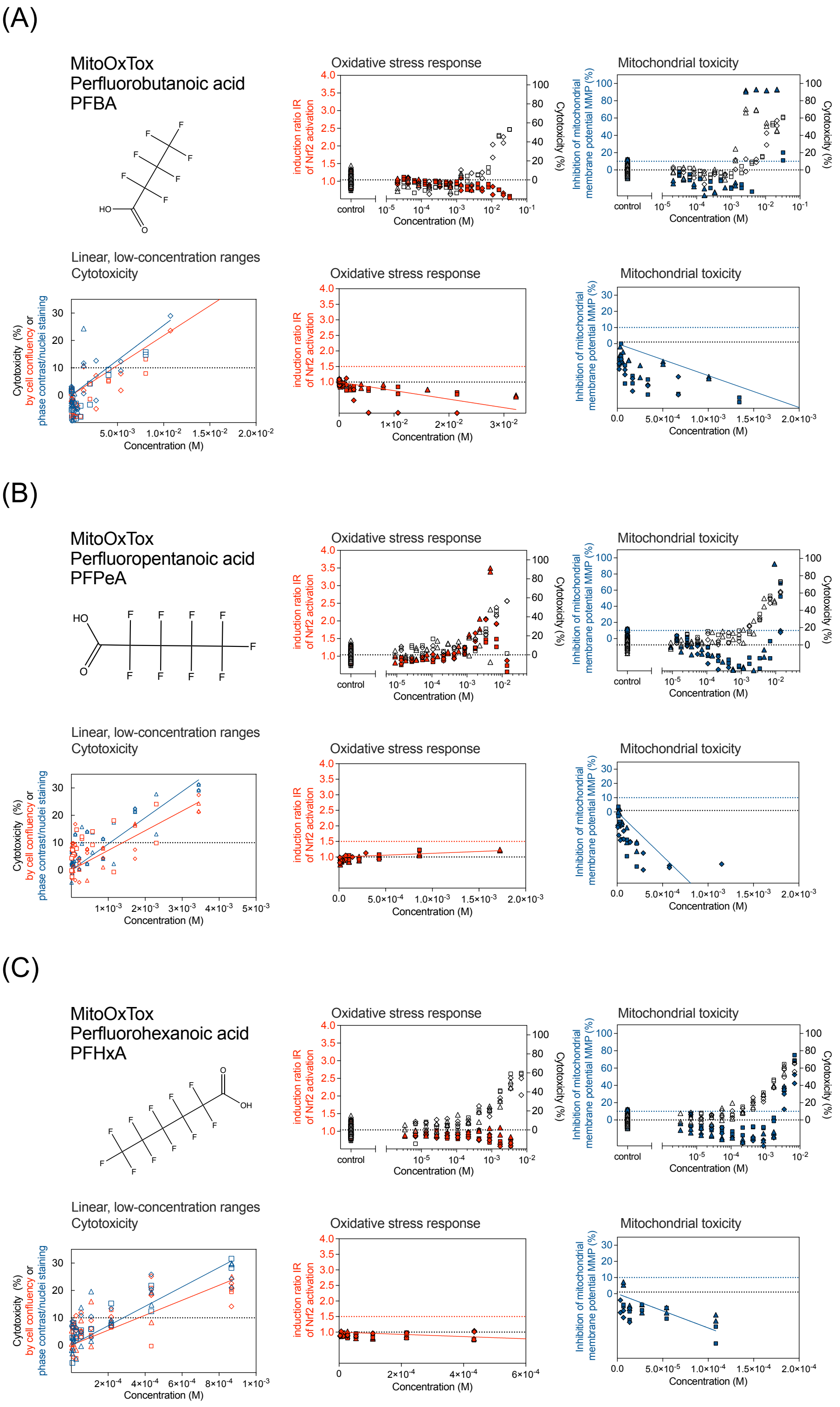

Figure S4. Concentration-response curves (CRC) of single PFAS in the MitoOxTox assay.

(D)

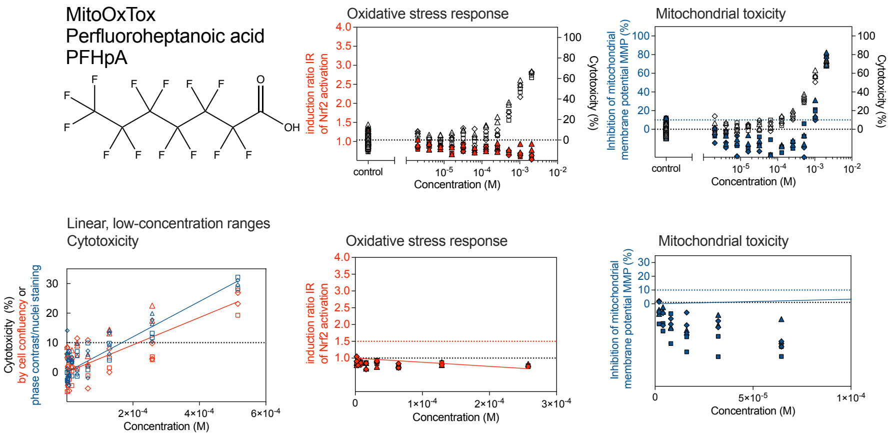

(E)

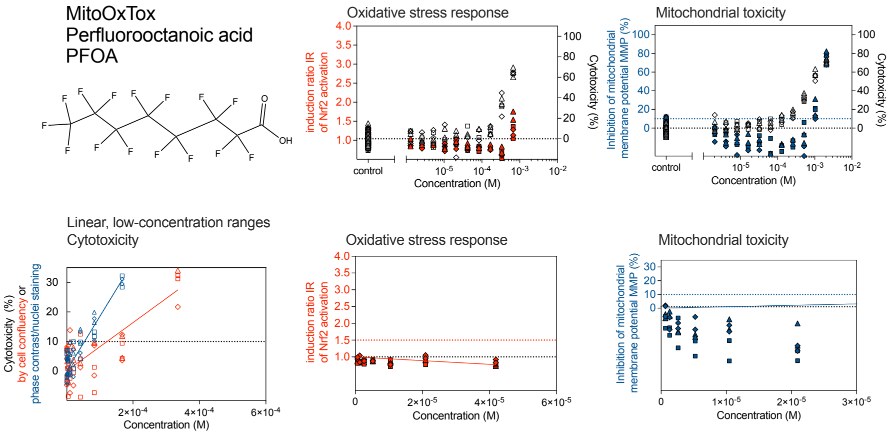

(F)

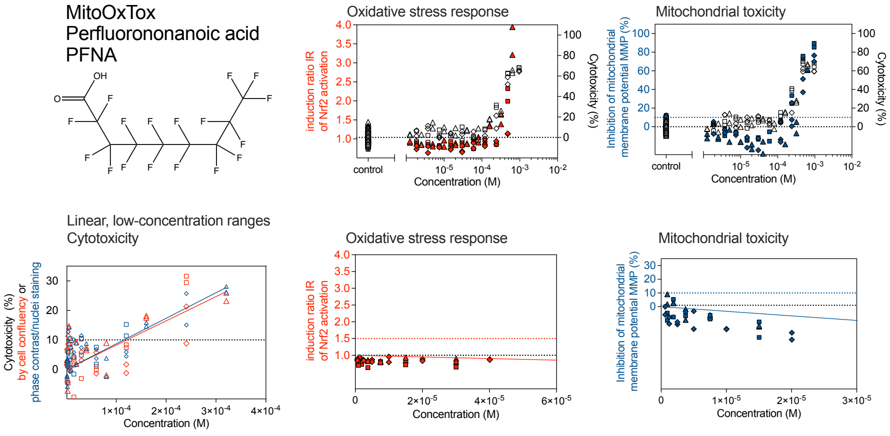

Figure S4 continued. Concentration-response curves (CRC) of single PFAS in the MitoOxTox assay.

(G)

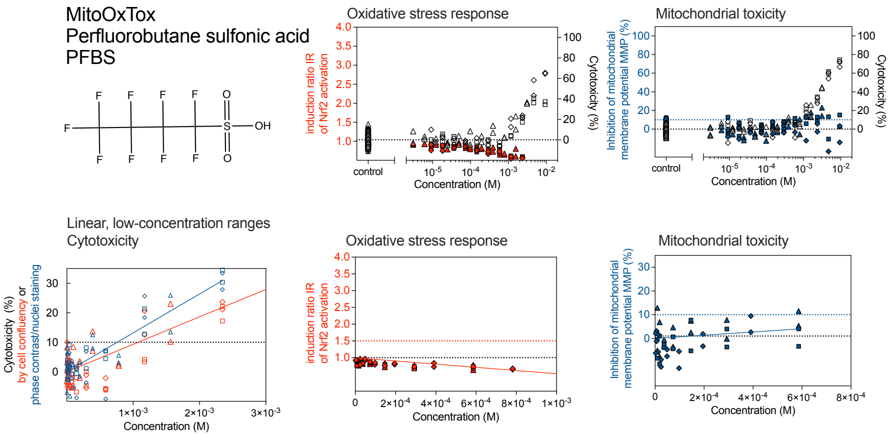

(H)

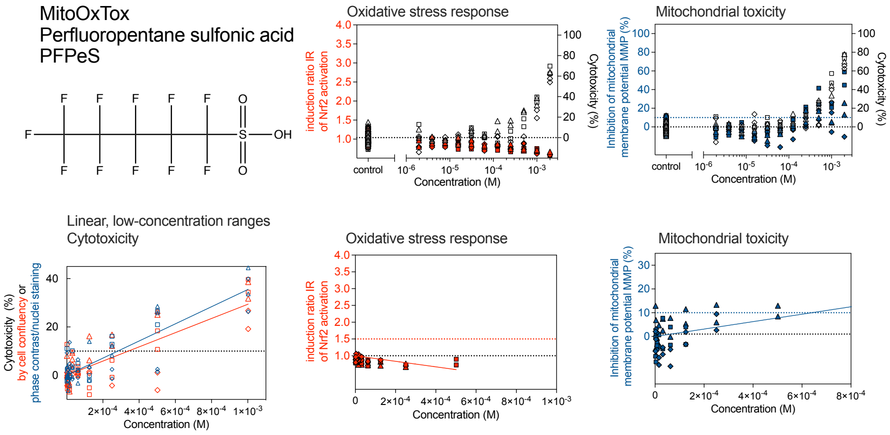

(I)

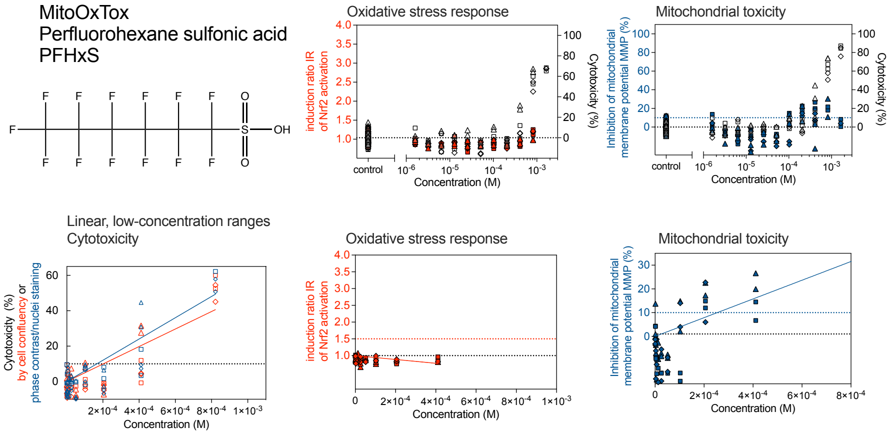

Figure S4 continued. Concentration-response curves (CRC) of single PFAS in the MitoOxTox assay.

(J)

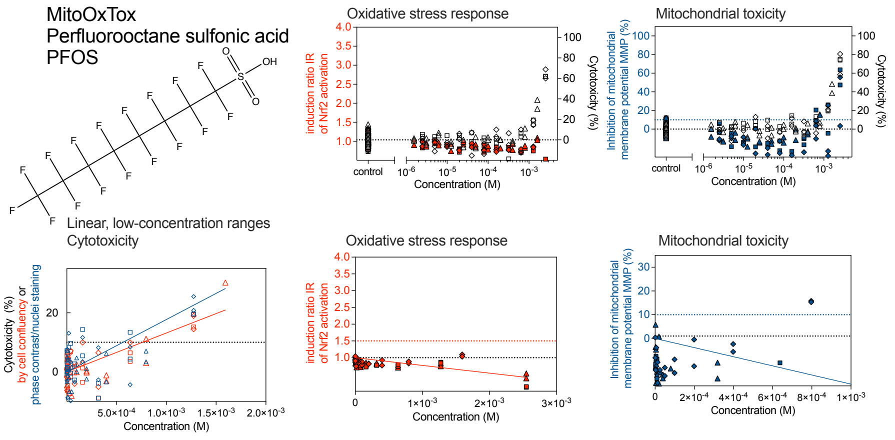

(K)

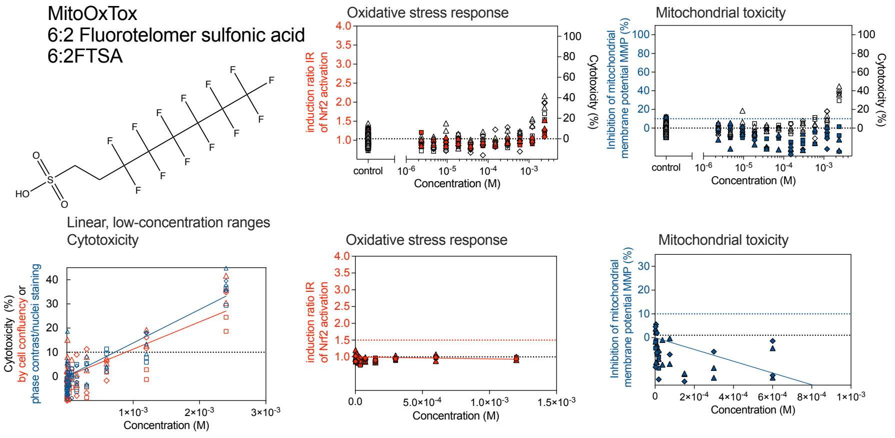

(L)

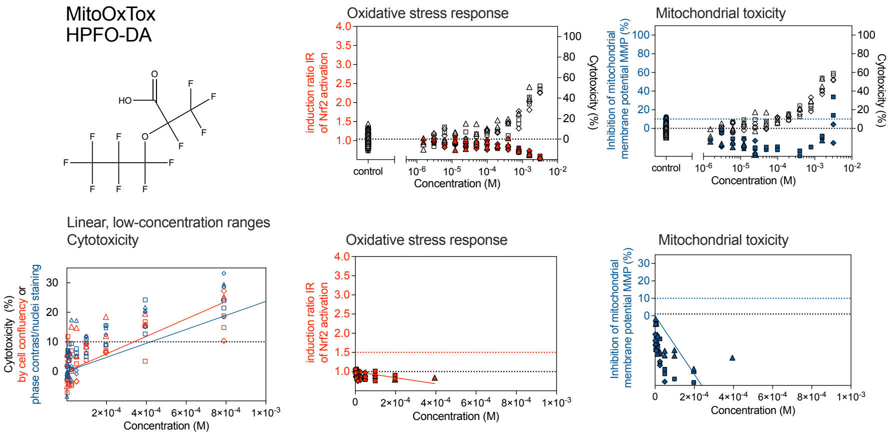

Figure S4 continued. Concentration-response curves (CRC) of single PFAS in the MitoOxTox assay. (A) PFBA, (B) PFPeA (C) PFHxA, (D) PFHpA, (E) PFOA, (F) PFNA, (G) PFBS, (H) PFPeS, (I) PFHxS, (J) PFOS, (K) 6:2 FTSA, (L) HFPO-DA. The top row depicts the CRCs for the oxidative stress response (activation of the Nrf2-ARE adaptive stress response pathway) and the mitochondrial toxicity (inhibition of the mitochondrial membrane potential MMP) on a logarithmic concentration scale to visualize all datapoints in relation to cytotoxicity. The bottom row only includes valid data in the linear range of the CRC up to 30% effect on a linear concentration scale and includes the fitted linear CRCs for cytotoxicity (left), oxidative stress

response (middle) and mitochondrial toxicity (right). Different symbols represent independent experiments.

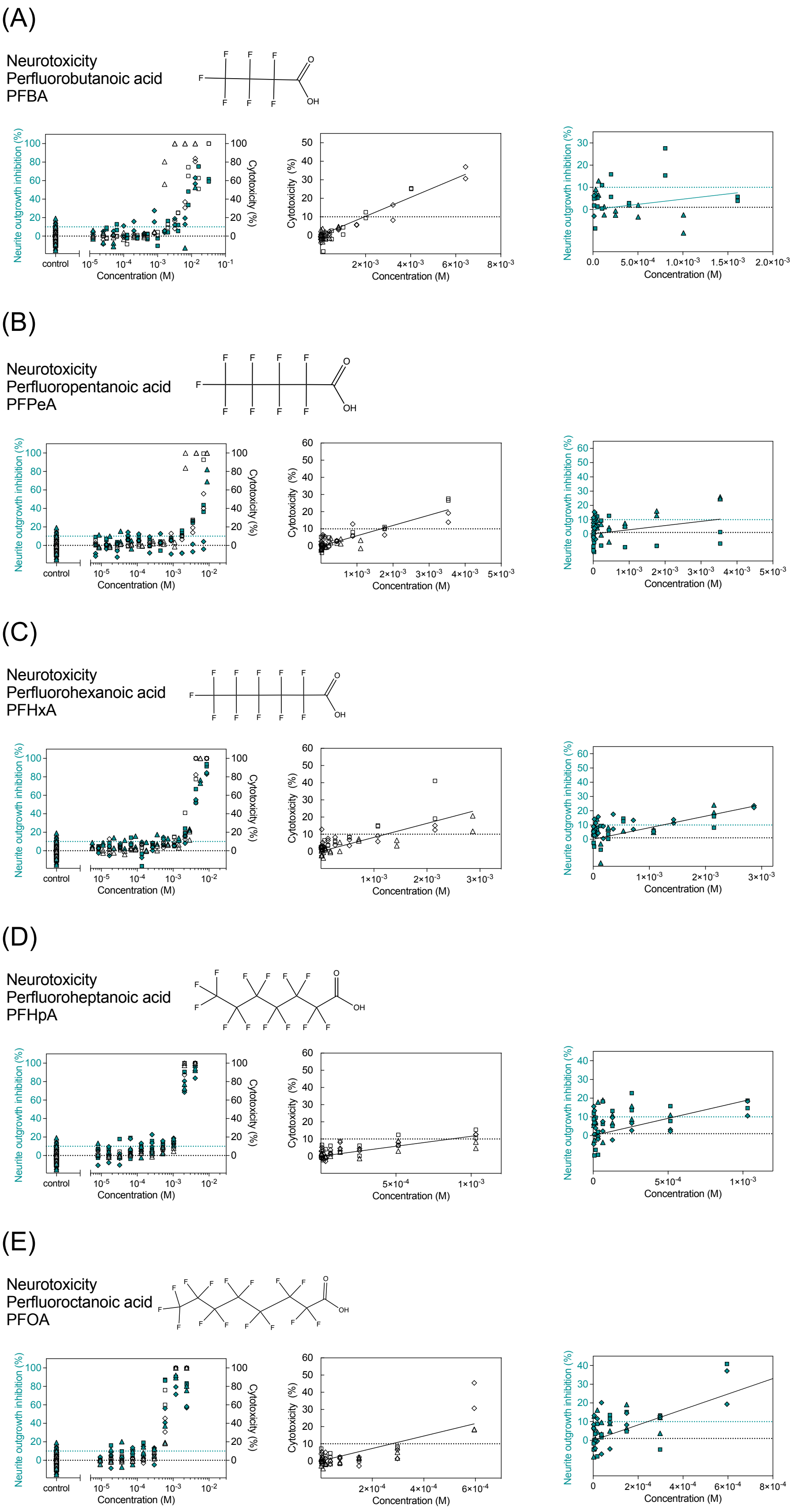

Figure S5. Concentration-response curves (CRC) of single PFAS in the neurotoxicity assay.

(F)

Neurotoxicity
Perfluorononanoic acid
PFNA

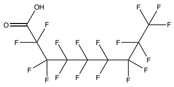

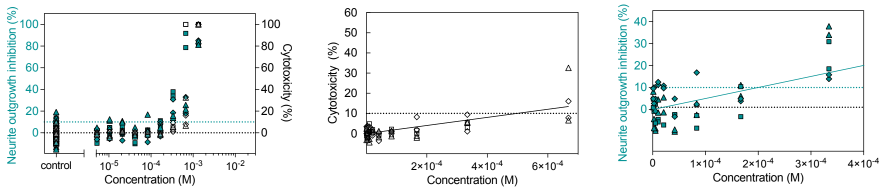

(G)

Neurotoxicity
Perfluorobutane sulfonic acid
PFBS

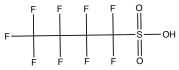

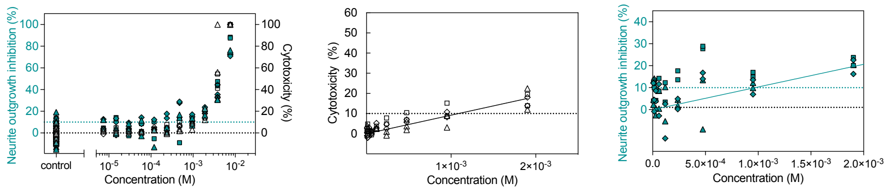

(H)

Neurotoxicity
Perfluoropentane sulfonic acid
PFPeS

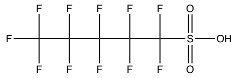

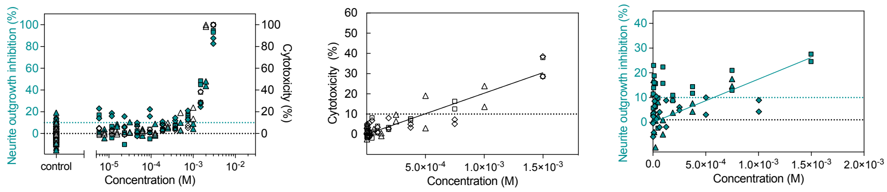

(I)

Neurotoxicity
Perfluorohexane sulfonic acid
PFHxS

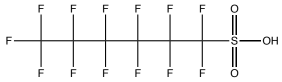

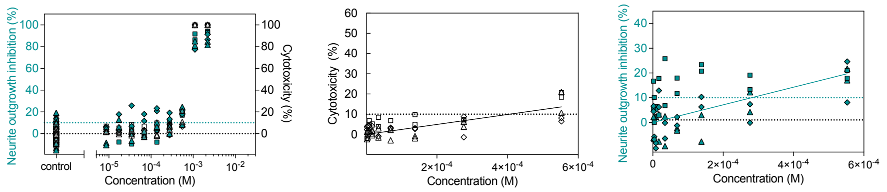

Figure S5 continued. Concentration-response curves (CRC) of single PFAS in the neurotoxicity assay.

(J)

Neurotoxicity
Perfluorooctane sulfonic acid
PFOS

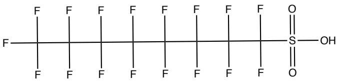

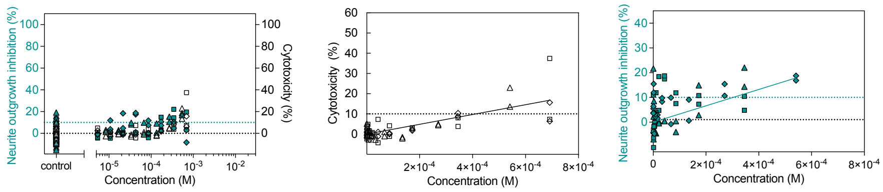

(K)

Neurotoxicity
6:2 Fluortelomer sulfonic acid
6:2 FTSA

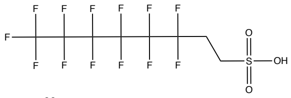

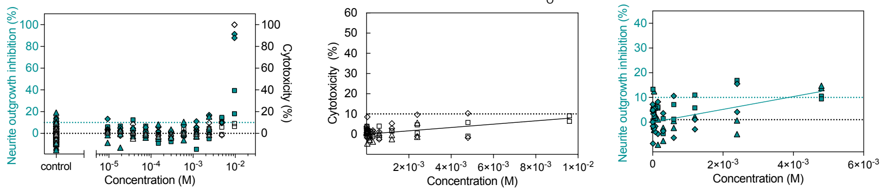

(L)

Neurotoxicity
2,3,3,3-Tetrafluoro-2-(heptafluoropropocy)propanoic acid
HFPO-DA

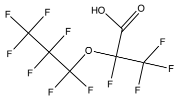

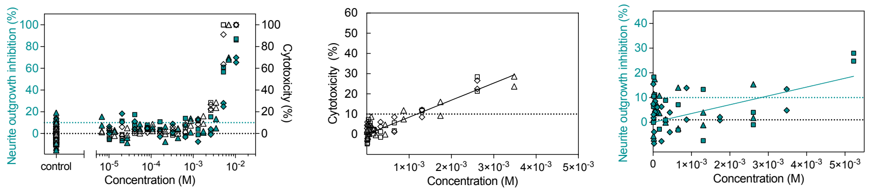

Figure S5 continued. Concentration-response curves (CRC) of single PFAS in the neurotoxicity assay. (A) PFBA, (B) PFPeA (C) PFHxA, (D) PFHpA, (E) PFOA, (F) PFNA, (G) PFBS, (H) PFPeS, (I) PFHxS, (J) PFOS, (K) 6:2 FTSA, (L) HFPO-DA. The left curve depicts the CRCs for the inhibition of neurite outgrowth (NOI) and cytotoxicity on a logarithmic concentration scale to visualize all datapoints. The middle plot only includes the linear range of the cytotoxicity CRC (<30% cytotoxicity) on a linear concentration scale and the line corresponds to the fitted linear CRCs for cytotoxicity. The right plot only includes the linear range of the NOI CRC (<30% NOI) on a linear concentration scale and the line corresponds to the fitted linear CRC for NOI. Different symbols represent independent experiments.

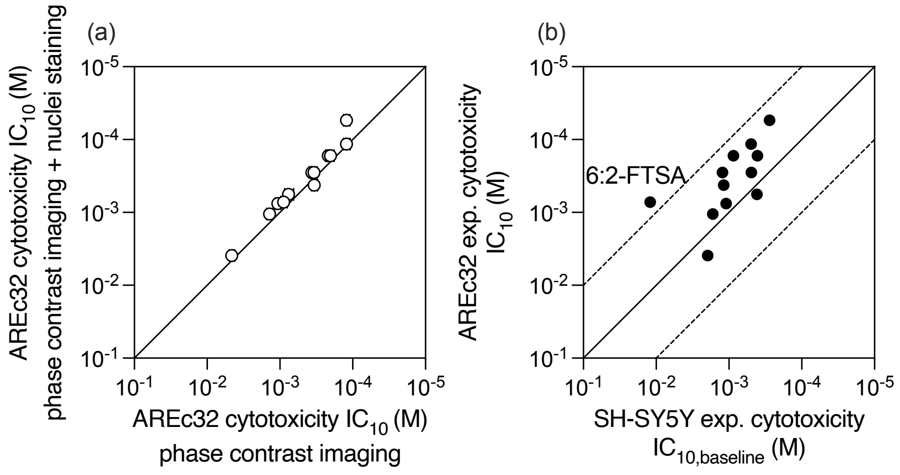

Figure S6. (a) Comparison of the cytotoxicity IC_{10} of AREc32 cells quantified with phase contrast imaging with and without nuclei staining. (b) Comparison of the cytotoxicity IC_{10} between the SH-SY5Y and AREc32 cells.

Text S7. Detailed discussion of the baseline toxicity analysis and comparison with literature data

The baseline toxicity QSARs of the anionic PFAS flatten at higher hydrophobicity due to their strong protein binding and are therefore distinctly different from the QSAR of the neutral PFAS.[8] The experimental data of the anionic PFAS included in this study aligned quite well with the prediction model for anionic PFAS (solid lines in Figure S7a) and with previous measurements.[8]

Despite the PFAS included here span more than four orders of magnitude in hydrophobicity, their cytotoxicity IC_{10} varied by less than two orders of magnitude (Figure S7). This is due to the strong binding to the medium proteins, which are mainly composed of albumins.[9] The AREc32 cell lines is supplemented with 10% FBS during the exposure experiment, which results in a protein content of 3.02 $mL_{protein}/mL_{medium}$ in the medium.[8] The neurobasal medium with supplements of SH-SY5Y also has a relatively high protein concentration of 2.38 $mL_{protein}/mL_{medium}$.[8] Binding to the proteins in the medium reduces the freely dissolved and hence bioavailable concentration of PFAS leading to an apparently lower sensitivity of the bioassays.[9] Despite the lower sensitivity, medium proteins stabilize the exposure system and deliver chemicals to the cells.[10] It was not attempted to minimize the experimental protein content as this would have led to other loss processes, such as binding to the well plates, and uptake into cells would have depleted the freely dissolved concentrations. This would have made the measurement of the freely dissolved concentrations necessary,[9] which is challenging for mixtures.

Figure S7 clearly illustrates the utility of the comparison with the baseline toxicity QSAR, not only for identification of specifically acting compounds (TR > 10) but also for metabolizable chemicals (TR < 0.1), such as 6:2 FTSA.[11] Apart from 6:2 FTSA, PFOS also had a low TR of 0.2, which is still within the validity range of baseline toxicity (0.1 < TR < 10) (Figure S7). PFOS is not metabolizable but can inhibit the activity of human cyp isoforms, which could be a cause of this deviation.[12]

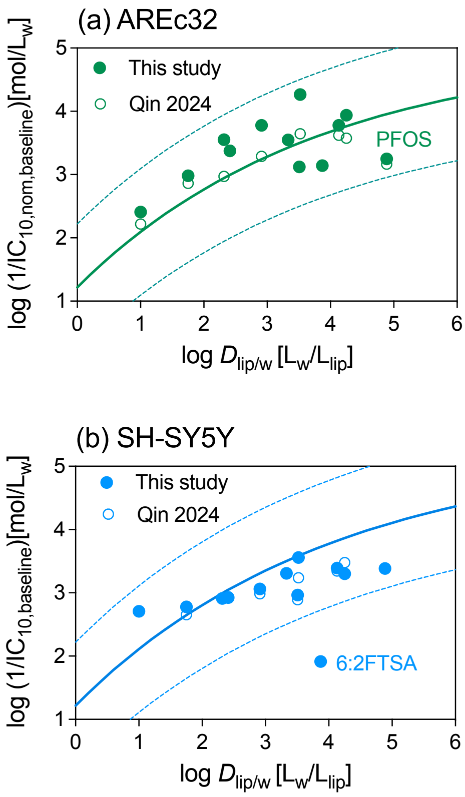

Figure S7. Measured inhibitory concentration causing 10% cytotoxicity IC_{10} of single chemicals for (a) AREc32 cells and (b) SH-SY5Y cells plotted against the hydrophobicity expressed as $\log D_{lip/w}$ and comparison with the baseline toxicity QSARs for anionic PFAS from Qin et al.[8] (eq. 6 for AREc32 and eq. 7 for SH-SY5Y, solid lines baseline toxicity QSAR, broken lines range of 0.1 < toxic ratio TR < 10.

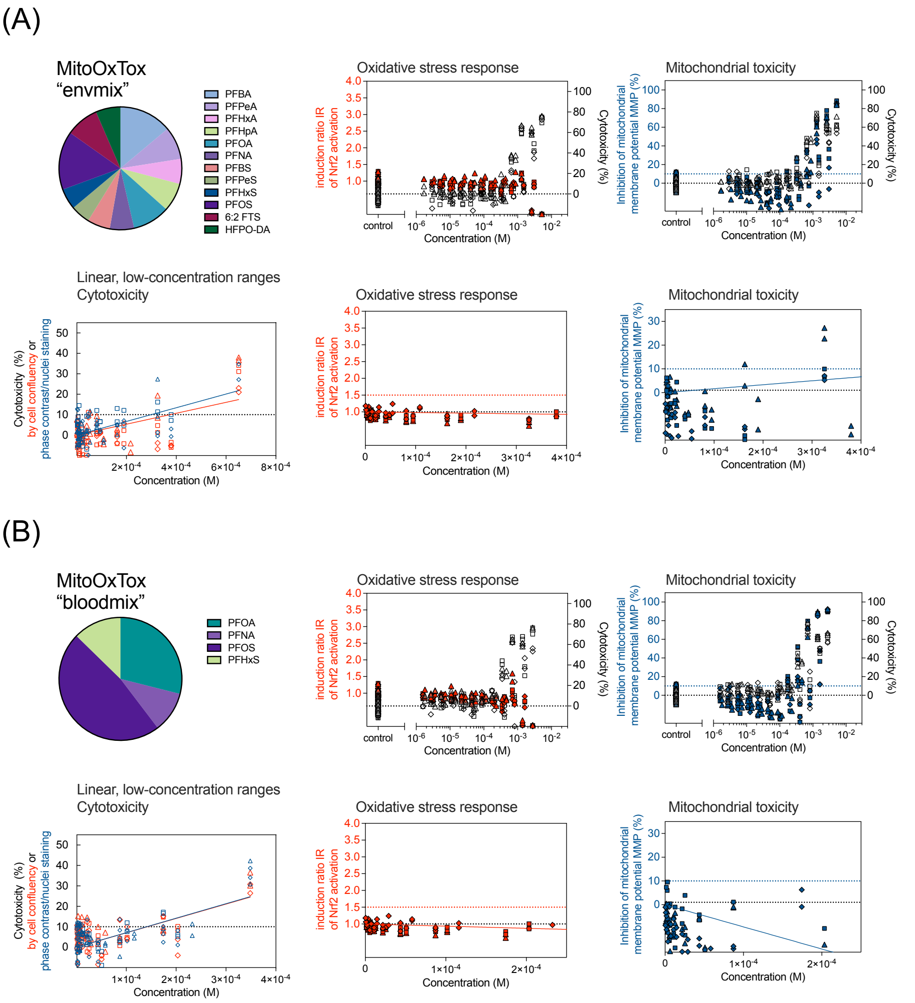

Figure S8. Top left: the pie chart represents the fraction p_i of the 12 PFAS (i) present in the mixture. Concentration-response curves of mixtures in the MitoOxTox assay. (A) envmix (B) bloodmix. The top row depicts the CRCs for the oxidative stress response (activation of the Nrf2-ARE adaptive stress response pathway) and the mitochondrial toxicity (inhibition of the mitochondrial membrane potential MMP) on a logarithmic concentration scale to visualize all datapoints in relation to cytotoxicity. The bottom row only includes valid data in the linear range of the CRC up to 30% effect on a linear concentration scale and includes the fitted linear CRCs for cytotoxicity (left), oxidative stress response (middle) and mitochondrial toxicity (right). Different symbols represent independent experiments.

(A)

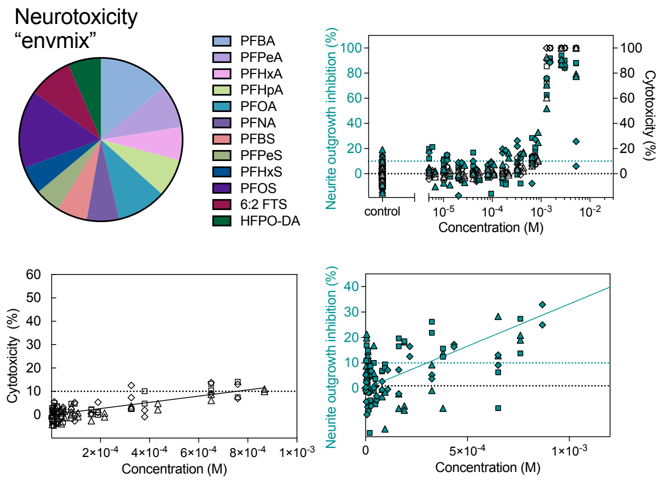

(B)

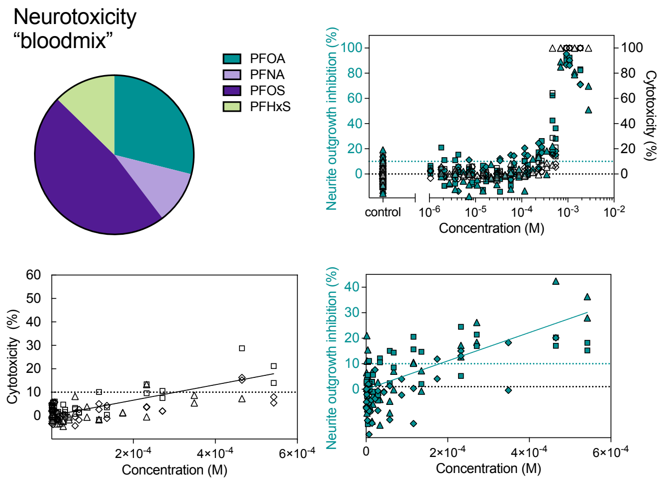

Figure S9. Concentration-response curves of mixture in the neurotoxicity assay: (A) envmix (B) bloodmix. Top left: the pie chart represents the fraction p_i of the 12 PFAS (i) present in the mixture. The top row depicts the CRCs on a logarithmic concentration scale to visualize all datapoints. The bottom row only includes valid data on a linear concentration scale and includes the fitted linear CRCs for each endpoint.

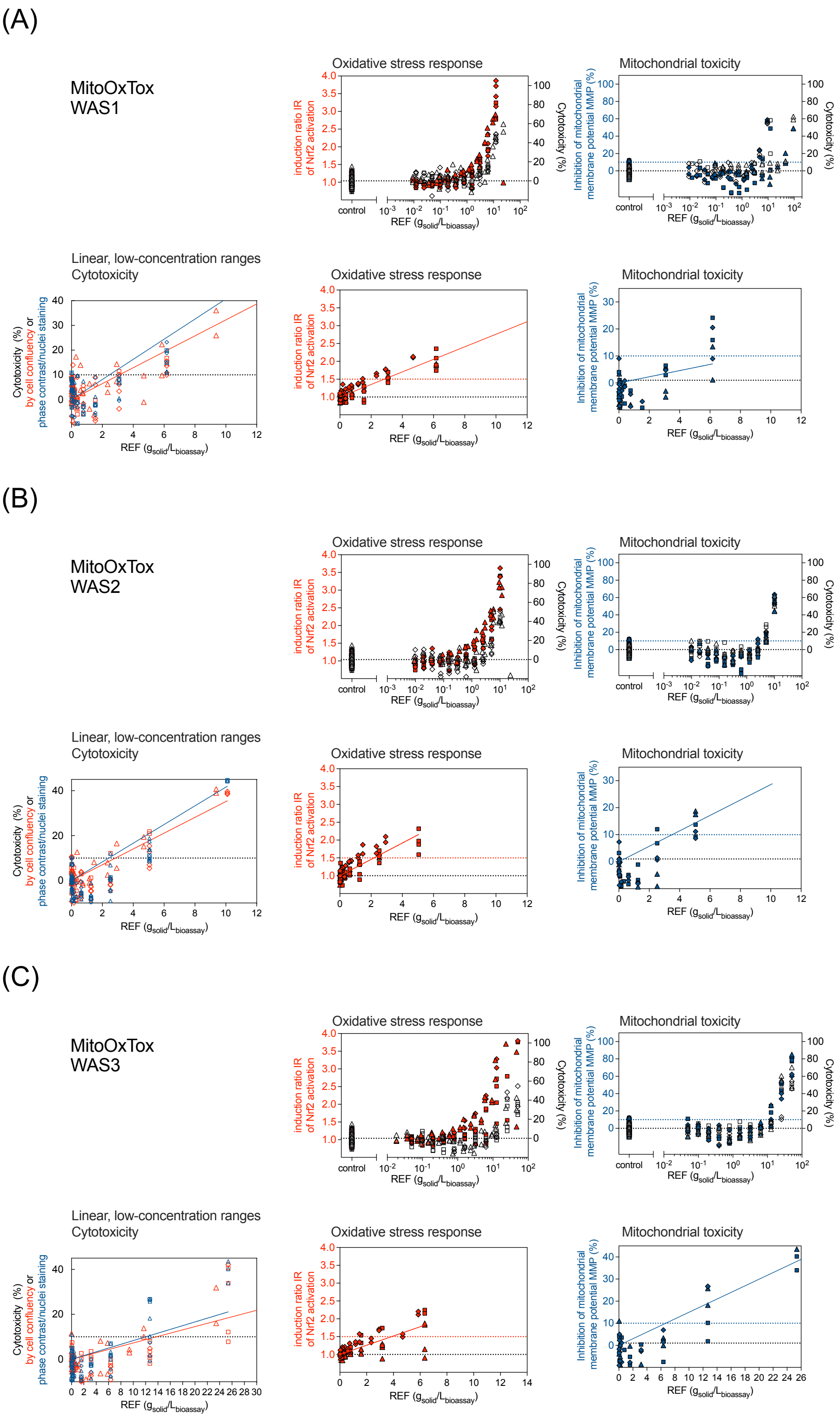

Figure S10. Concentration-response curves of extracts from (A-C) wastewater (WAS) and (D-E) primary biosolids (PS) in the MitoOxTox assay.

(D)

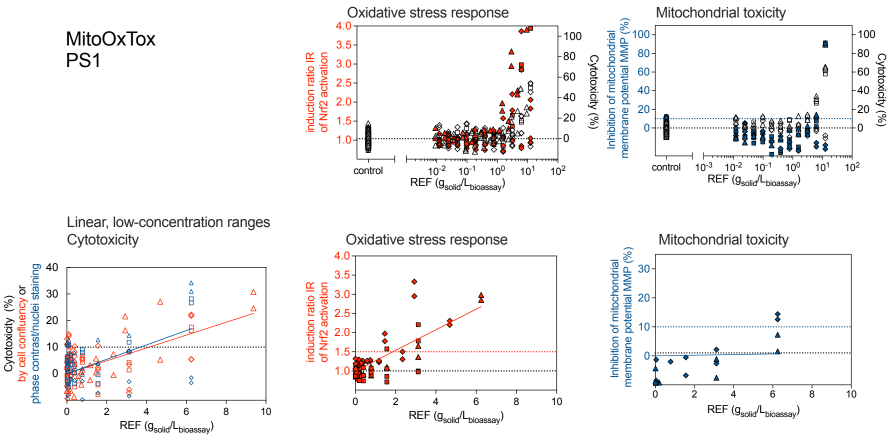

(E)

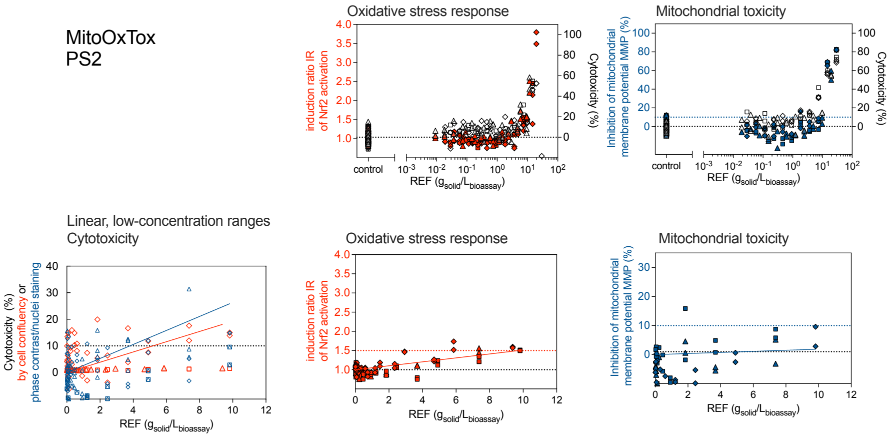

(F)

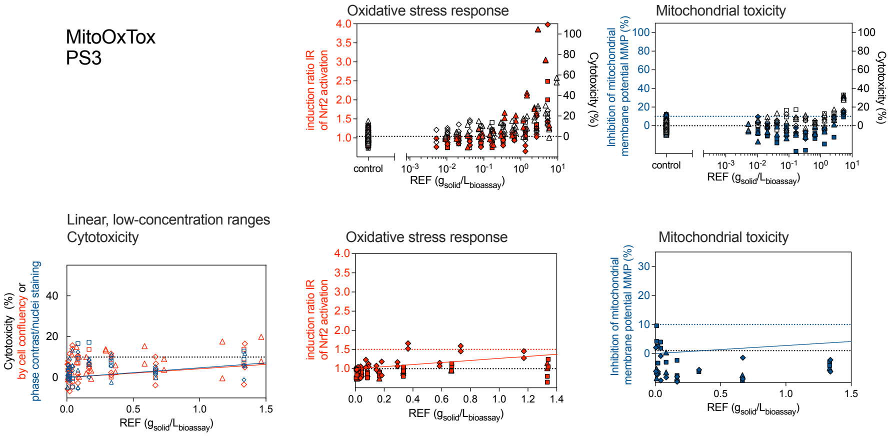

Figure S10 continued. Concentration-response curves of extracts from (A-C) wastewater (WAS) and (D-E) primary biosolids (PS) in the MitoOxTox assay. The dosed concentrations are given as relative enrichment factor REF in units of $g_{solid}/L_{bioassay}$. The top row depicts the CRCs for the oxidative stress response (activation of the Nrf2-ARE adaptive stress response pathway) and the mitochondrial toxicity (inhibition of the mitochondrial membrane potential MMP) on a logarithmic concentration scale to visualize all datapoints in relation to cytotoxicity. The bottom row only includes valid data in the linear range of the CRC up to 30% effect on a linear concentration scale and includes the fitted linear CRCs for cytotoxicity (left), oxidative

stress response (middle) and mitochondrial toxicity (right). Different symbols represent independent experiments

(A)

Neurotoxicity WAS1

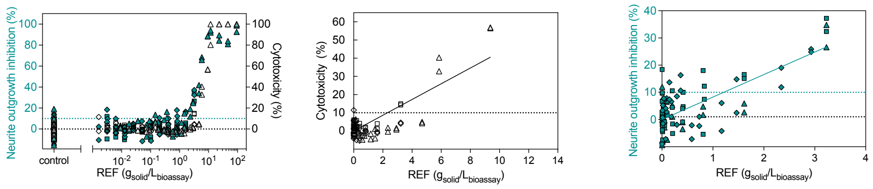

(B)

Neurotoxicity WAS2

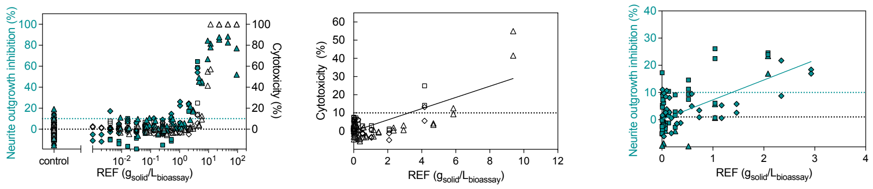

(C)

Neurotoxicity WAS3

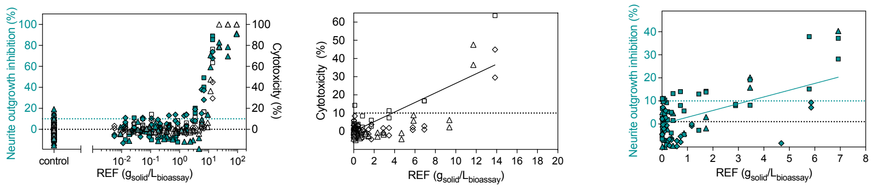

(D)

Neurotoxicity PS1

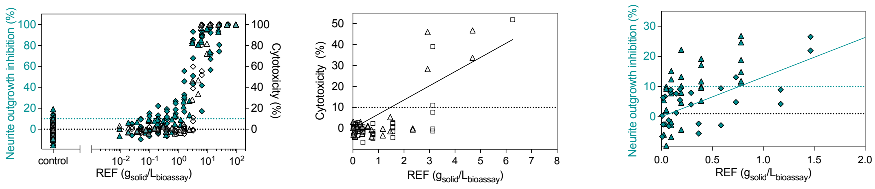

(E)

Neurotoxicity PS2

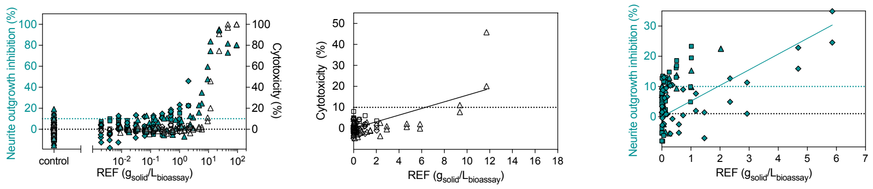

Figure S11. Concentration-response curves of extracts from (A-C) wastewater activated sludge (WAS) and (D-E) primary biosolids (PS) in the neurotoxicity assay.

(F)

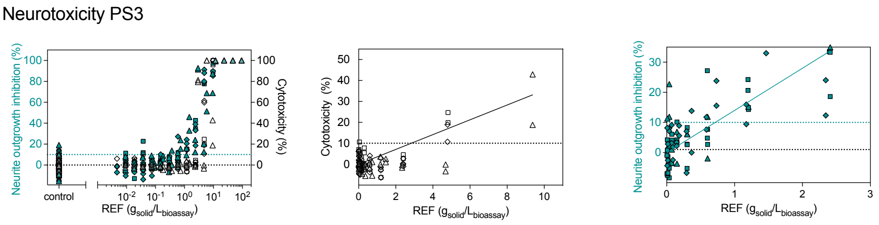

Figure S11 continued. Concentration-response curves of extracts from (A-C) wastewater activated sludge (WAS) and (D-E) primary biosolids (PS) in the neurotoxicity assay. The dosed concentrations are given as relative enrichment factor REF in units of $g_{solid}/L_{bioassay}$. The left curve depicts the CRCs for the inhibition of neurite outgrowth (NOI) and cytotoxicity on a logarithmic concentration scale to visualize all datapoints. The middle plot only includes the linear range of the cytotoxicity CRC (<30% cytotoxicity) on a linear concentration scale and the line corresponds to the fitted linear CRCs for cytotoxicity. The right plot only includes the linear range of the NOI CRC (<30% NOI) on a linear concentration scale and the line corresponds to the fitted linear CRC for NOI. Different symbols represent independent experiments.

Table S1. Effect concentrations $EC_{IR1.5}$ for activation of oxidative stress response, EC_{10} for inhibition of the mitochondrial membrane potential (MMP), cytotoxicity inhibitory concentrations IC_{10} for AREc32 and SH-SY5Y cells and effect concentration EC_{10} for 10% reduction of neurite length by the sample extracts. The concentrations are relative enrichment factors REF in units of $g_{solid}/L_{bioassay}$.

		Wastewater activated sludge (WAS)			Primary solid (PS)		
Bioassay endpoints	Units of REF ($g_{solid}/L_{bioassay}$)	EC	SD	CV%	EC	SD	CV%
AREc32 activation	$EC_{IR1.5}$	4.49	4.54	101%	2.96	0.85	28.6%
AREc32 MMP inhibition	EC_{10}	1.42	1.76	124%	0.32	0.13	41.8%
AREc32 cytotoxicity	IC_{10}	3.88	1.44	37.2%	6.20	6.54	105%
SH-SY5Y cytotoxicity	IC_{10}	3.54	2.50	70.6%	3.12	0.75	24.1%
SH-SY5Y NOI	EC_{10}	1.14	0.69	60.7%	2.00	1.23	61.4%

(A)

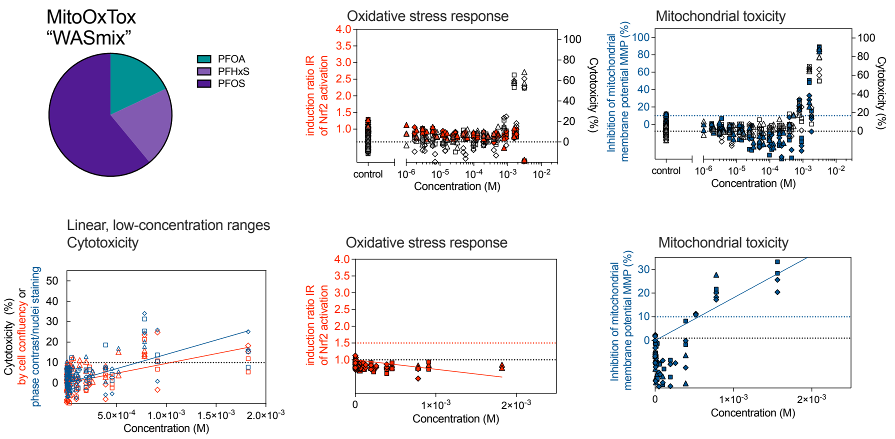

(B)

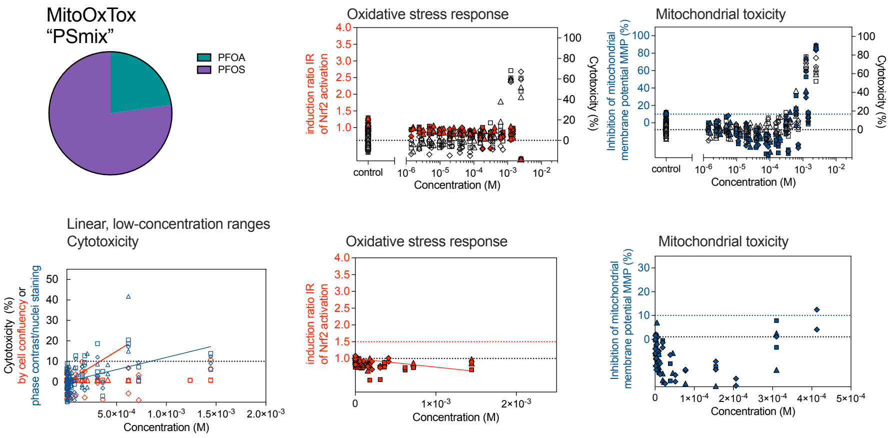

Figure S12. Pie chart represents the fraction p_i of the two or three PFAS (i) present in the mixture. Concentration-response curves of the designed wastewater mixtures in the MitoOxTox assay. (A) WASmix, (B) PSmix. . The top row depicts the CRCs for the oxidative stress response (activation of the Nrf2-ARE adaptive stress response pathway) and the mitochondrial toxicity (inhibition of the mitochondrial membrane potential MMP) on a logarithmic concentration scale to visualize all datapoints in relation to cytotoxicity. The bottom row only includes valid data in the linear range of the CRC up to 30% effect on a linear concentration scale and includes the fitted linear CRCs for cytotoxicity (left), oxidative stress response (middle) and mitochondrial toxicity (right). Different symbols represent independent experiments.

(A)

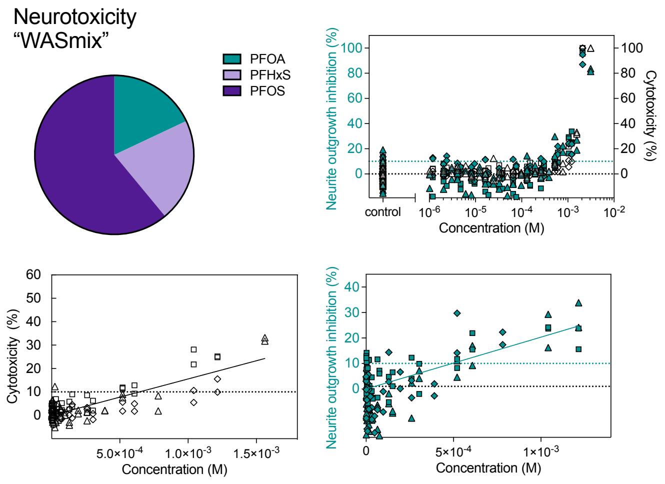

(B)

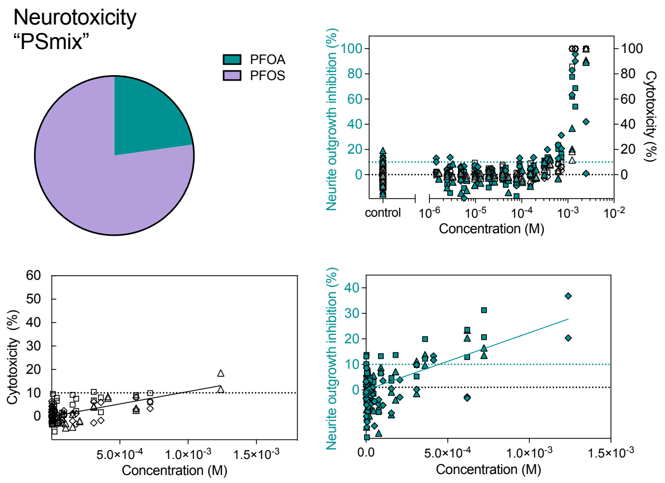

Figure S13. Pie chart represents the fraction pi of the two or three PFAS (i) present in the mixture. Concentration-response curves of the designed wastewater mixtures in the neurotoxicity assay. (A) WASmix, (B) PSmix. The left curve depicts the CRCs for the inhibition of neurite outgrowth (NOI) and cytotoxicity on a logarithmic concentration scale to visualize all datapoints. The middle plot only includes the linear range of the cytotoxicity CRC (<30% cytotoxicity) on a linear concentration scale and the line corresponds to the fitted linear CRCs for cytotoxicity. The right plot only includes the linear range of the NOI CRC (<30% NOI) on a linear concentration scale and the line corresponds to the fitted linear CRC for NOI. Different symbols represent independent experiments.

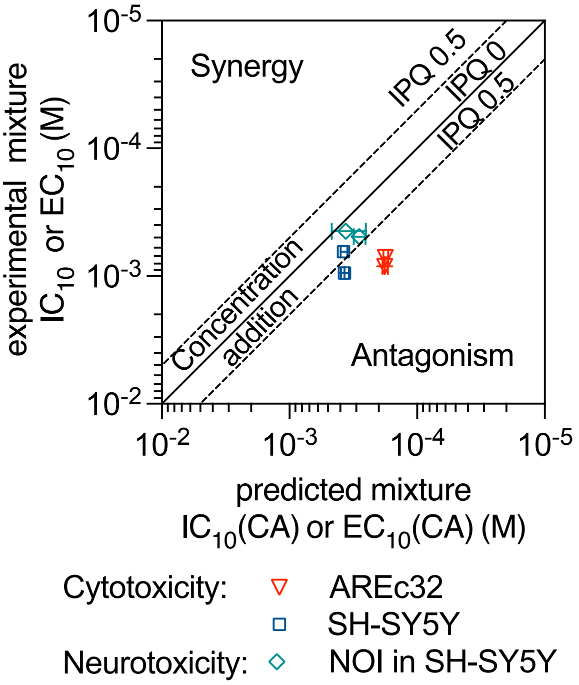

Figure S14. Comparison between the experimental mixture IC_{10} (inhibitory concentration causing 10% cytotoxicity) with the predicted mixture IC_{10}(CA) calculated with the mixture model of concentration addition (CA) (eq 8-10) for AREc32 and SH-SY5Y cells for the WASmix and PSmix; comparison of experimental and predicted EC_{10} (effect concentration causing 10% reduction of neurite length) for neurite outgrowth inhibition (NOI) in SH-SY5Y. The line corresponds to perfect agreement between model and prediction (index of prediction quality (e. 10) IPQ = 1), the broken lines mark the area of IPQ up to 0.5. No data lay in the upper left corner, where synergistic effects would be displayed but the cytotoxicity data showed a tendency towards antagonistic effects.

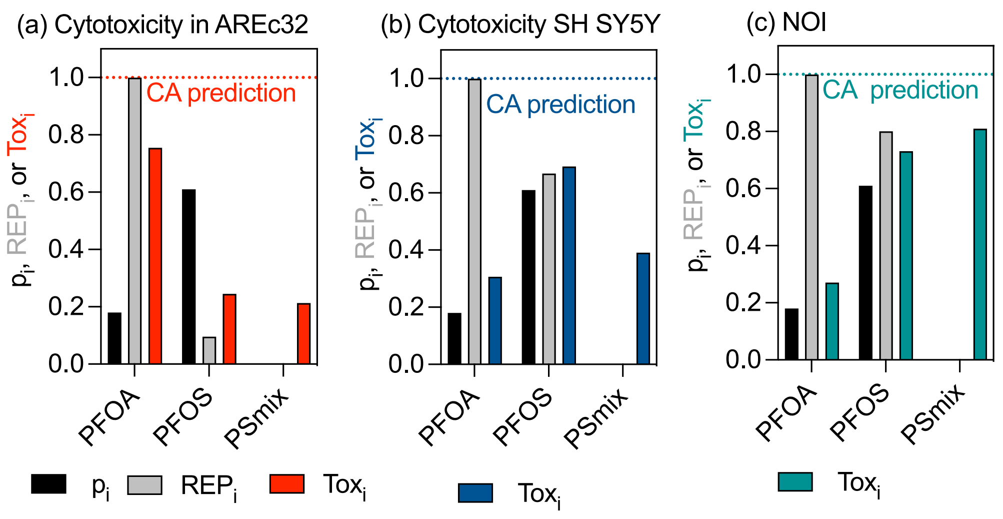

Figure S15. PSmix: Comparison of contribution of individual PFAS to the fraction in the mixture (p_i), their relative effect potency compared to PFOA (REP_i= $IC_{10,PFOA}/IC_{10,i}$ or $EC_{10,PFOA}/EC_{10,i}$) and their contribution to the mixture toxicity (Tox_i, eq. 13). (A) cytotoxicity in AREc32, (B) cytotoxicity in SH SY5Y, (C) neurite outgrowth inhibition (NOI) in SH SY5Y.

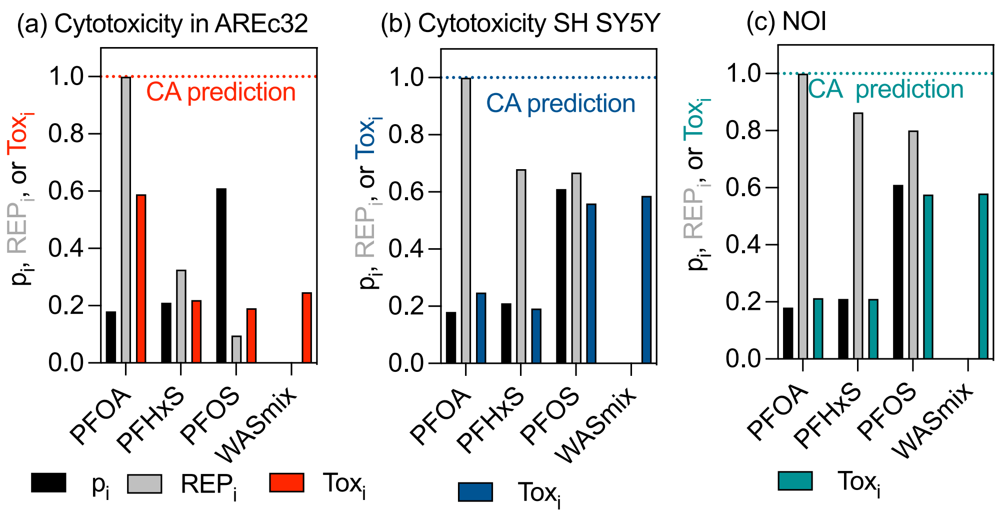

Figure S16. WASmix: Comparison of contribution of individual PFAS to the fraction in the mixture (p_i), their relative effect potency compared to PFOA (REP_i= $IC_{10,PFOA}/IC_{10,i}$ or $EC_{10,PFOA}/EC_{10,i}$) and their contribution to the mixture toxicity (Tox_i, eq. 13). (A) cytotoxicity in AREc32, (B) cytotoxicity in SH SY5Y, (C) neurite outgrowth inhibition (NOI) in SH SY5Y.

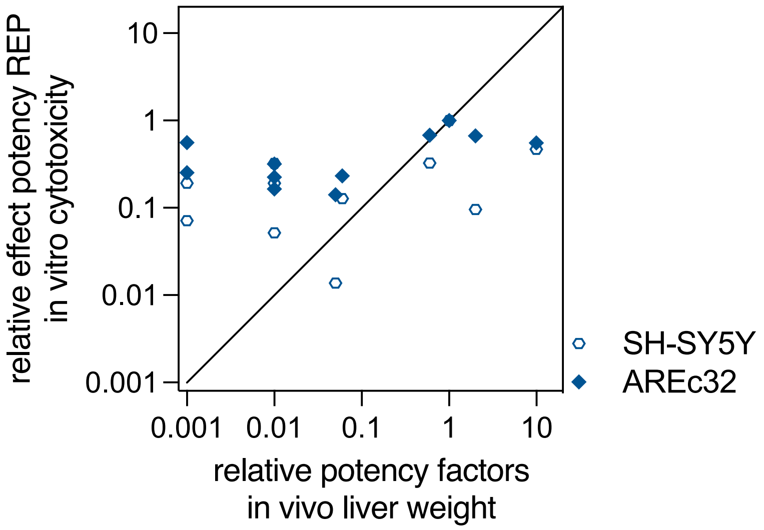

Figure S17. Comparison of in vitro REP of cytotoxicity and relative potency factors in vivo for liver toxicity (weight gain) on male rats that were orally dosed for 42 to 90 days.[13]

Table S2. Effects of mixture components expressed as PFOA equivalent concentrations (PFOA-EQ) of the environmental mixture (envmix) and the blood mixture (blood mix).

	Environmental mixture (envmix)			Blood mixture (bloodmix)		
Abbreviation	PFOA-EQ$_{chem,i}$ (ng/L) cytotoxicity AREc32	PFOA-EQ$_{chem,i}$ (ng/L) cytotoxicity SH SY5Y	PFOA-EQ$_{chem,i}$ (ng/L) NOI	PFOA-EQ$_{chem,i}$ (ng/L) cytotoxicity AREc32	PFOA-EQ$_{chem,i}$ (ng/L) cytotoxicity SH SY5Y	PFOA-EQ$_{chem,i}$ (ng/L) NOI
PFBA	0.2	2.2	1.8			
PFPeA	0.5	1.6	0.7			
PFHxA	1.4	1.7	1.5			
PFHpA	2.7	2.7	3.8			
PFOA	11.0	11.0	11.0	2.0	2.0	2.0
PFNA	3.4	4.0	8.7	0.3	0.4	0.9
PFBS	0.5	1.7	1.7			
PFPeS	1.1	3.2	2.4			
PFHxS	2.0	4.1	5.3	0.3	0.6	0.7
PFOS	1.7	11.5	13.8	0.3	2.2	2.6
6:2 FTS	0.7	0.2	0.6			
HPFO-DA	0.9	1.7	0.6			
PFOA-EQ$_{chem}$ (ng/L)	26.1	45.5	51.7	2.9	5.1	6.2
PFOA-EQ$_{bio, mix}$ (ng/L) designed mixture	20.3	40.9	89.9	2.6	6.2	9.1

References

(1) Lee, J.; König, M.; Braun, G.; Escher, B. I. Water Quality Monitoring with the Multiplexed Assay MitoOxTox for Mitochondrial Toxicity, Oxidative Stress Response, and Cytotoxicity in AREc32 Cells. *Environ. Sci. Technol.* **2024**, *58* (13), 5716-5726. DOI: 10.1021/acs.est.3c09844.

(2) Stringer, C.; Wang, T.; Michaelos, M.; Pachitariu, M. Cellpose: a generalist algorithm for cellular segmentation. *Nat. Methods* **2021**, *18* (1), 100-+. DOI: 10.1038/s41592-020-01018-x.

(3) Stirling, D. R.; Swain-Bowden, M. J.; Lucas, A. M.; Carpenter, A. E.; Cimini, B. A.; Goodman, A. CellProfiler 4: improvements in speed, utility and usability. *Bmc Bioinformatics* **2021**, *22* (1). DOI: 10.1186/s12859-021-04344-9.

(4) Sakamuru, S.; Li, X.; Attene-Ramos, M. S.; Huang, R. L.; Lu, J. M.; Shou, L.; Shen, M.; Tice, R. R.; Austin, C. P.; Xia, M. H. Application of a homogenous membrane potential assay to assess mitochondrial function. *Physiological Genomics* **2012**, *44* (9), 495-503. DOI: 10.1152/physiolgenomics.00161.2011.

(5) Escher, B. I.; Dutt, M.; Maylin, E.; Tang, J. Y. M.; Toze, S.; Wolf, C. R.; Lang, M. Water quality assessment using the AREc32 reporter gene assay indicative of the oxidative stress response pathway. *J. Environ. Monitor.* **2012**, *14* (11), 2877-2885. DOI: 10.1039/c2em30506b.

(6) Escher, B.; Neale, P. A.; Villeneuve, D. The advantages of linear concentration-response curves for *in vitro* bioassays with environmental samples. *Environ. Toxicol. Chem.* **2018**, *37* (9), 2273–2280. DOI: 10.1002/etc.4178.

(7) Lee, J.; Escher, B. I.; Scholz, S.; Schlichting, R. Inhibition of neurite outgrowth and enhanced effects compared to baseline toxicity in SH-SY5Y cells. *Arch. Toxicol.* **2022**, *96*, 1039–1053. DOI: 10.1007/s00204-022-03237-x.

(8) Qin, W.; Henneberger, L.; Glüge, J.; König, M.; Escher, B. I. Baseline Toxicity Model to Identify the Specific and Nonspecific Effects of Per- and Polyfluoroalkyl Substances in Cell-Based Bioassays. *Environ. Sci. Technol.* **2024**, *58* (13), 5727-5738. DOI: 10.1021/acs.est.3c09950.

(9) Qin, W.; Henneberger, L.; Huchthausen, J.; König, M.; Escher, B. I. Role of bioavailability and protein binding of four anionic perfluoroalkyl substances in cell-based bioassays for quantitative in vitro to in vivo extrapolations. *Environ. Internat.* **2023**, *173*, 107857. DOI: 10.1016/j.envint.2023.107857.

(10) Fischer, F. C.; Henneberger, L.; Schlichting, R.; Escher, B. I. How To Improve the Dosing of Chemicals in High-Throughput in Vitro Mammalian Cell Assays. *Chem. Res. Toxicol.* **2019**, *32* (8), 1462-1468. DOI: 10.1021/acs.chemrestox.9b00167.

(11) Huchthausen, J.; Braasch, J.; Escher, B. I.; König, M.; Henneberger, L. Effects of Chemicals in Reporter Gene Bioassays with Different Metabolic Activities Compared to

Baseline Toxicity. *Chem. Res. Toxicol.* **2024**, *37* (5), 744-756. DOI: 10.1021/acs.chemrestox.4c00017.

(12) Narimatsu, S.; Nakanishi, R.; Hanioka, N.; Saito, K.; Kataoka, H. Characterization of inhibitory effects of perfluorooctane sulfonate on human hepatic cytochrome P450 isoenzymes: Focusing on CYP2A6. *Chem.-Biol. Interact.* **2011**, *194* (2), 120-126. DOI: 10.1016/j.cbi.2011.09.002.

(13) Bil, W.; Zeilmaker, M.; Fragki, S.; Lijzen, J.; Verbruggen, E.; Bokkers, B. Risk Assessment of Per- and Polyfluoroalkyl Substance Mixtures: A Relative Potency Factor Approach. *Environ. Toxicol. Chem.* **2022**. DOI: 10.1002/etc.4835.
